# Novel hybridization- and tag-based error-corrected method for sensitive ctDNA mutation detection using ion semiconductor sequencing

**DOI:** 10.1038/s41598-022-09698-5

**Published:** 2022-04-06

**Authors:** Kjersti Tjensvoll, Morten Lapin, Bjørnar Gilje, Herish Garresori, Satu Oltedal, Rakel Brendsdal Forthun, Anders Molven, Yves Rozenholc, Oddmund Nordgård

**Affiliations:** 1 Department of Hematology and Oncology, Laboratory for Molecular Biology, Stavanger University Hospital, 4068 Stavanger, Norway; 2 BioSTM UR 7537, Faculté de Pharmacie de Paris, Université Paris Citè, 75006 Paris, France; 3 Department of Medical Genetics, Haukeland University Hospital, 5020 Bergen, Norway; 4 Department of Internal Medicine, Hematology Section, Haukeland University Hospital, 5020 Bergen, Norway; 5 Gade Laboratory for Pathology, Department of Clinical Medicine, University of Bergen, 5020 Bergen, Norway; 6 Department of Pathology, Haukeland University Hospital, 5021 Bergen, Norway

**Keywords:** Tumour biomarkers, Molecular biology

## Abstract

Circulating tumor DNA (ctDNA) analysis has emerged as a clinically useful tool for cancer diagnostics and treatment monitoring. However, ctDNA detection is complicated by low DNA concentrations and technical challenges. Here we describe our newly developed sensitive method for ctDNA detection on the Ion Torrent sequencing platform, which we call HYbridization- and Tag-based Error-Corrected sequencing (HYTEC-seq). This method combines hybridization-based capture with molecular tags, and the novel variant caller PlasmaMutationDetector2 to eliminate background errors. We describe the validation of HYTEC-seq using control samples with known mutations, demonstrating an analytical sensitivity down to 0.1% at > 99.99% specificity. Furthermore, to demonstrate the utility of this method in a clinical setting, we analyzed plasma samples from 44 patients with advanced pancreatic cancer, revealing mutations in 57% of the patients at allele frequencies as low as 0.23%.

## Introduction

Circulating tumor DNA (ctDNA) is cell-free DNA (cfDNA) that is shed into the bloodstream from apoptotic or necrotic tumor cells, and thus reflects the genomic features of the tumor of origin^1^. Since ctDNA originates from dying cells in the primary tumor or metastatic lesions, or from lysis of circulating tumor cells, ctDNA analysis represents a non-invasive method of assessing a cancer patient’s total tumor burden^1^. Notably, ctDNA assessment may reveal sub-clonal mutations that are missed by tumor biopsy analysis due to intra- and inter-tumor heterogeneity, and can also be used for detection of cancer recurrence, and monitoring of disease and therapy response^2,3^. However, ctDNA usually comprises only a small fraction of the total cfDNA content in the circulation, as cfDNA is also released from normal cells. Therefore, sensitive detection methods are needed for ctDNA analysis to be clinically useful.

Tumor-specific mutations are commonly utilized as surrogate markers for ctDNA detection, and studies demonstrate the clinical relevance of this approach for ctDNA detection in several cancer types^4–7^. Moreover, the use of large gene panels and high-throughput sequencing can facilitate extensive ctDNA characterization, thereby enabling personalized cancer profiling^8–10^. However, sequencing-based ctDNA detection is hampered by a relatively high rate of PCR and sequencing errors, which limit the analytical specificity^11^. Sequencing approaches using molecular tags—also called unique molecular identifiers (UMIs)—have enabled the tracking of individual ctDNA fragments, and thus improved discrimination of true mutations from errors/artifacts^10–12^. In recent years, error suppression based on error profiling in normal control samples has further improved the sensitivity for detection of low-frequency ctDNA mutations^13,14^.

Here we present a new and highly sensitive sequencing method for Ion Torrent sequencing chemistry, called HYbridization- and Tag-based Error-Corrected sequencing (HYTEC-seq). This new method combines hybridization-based target capture with advanced error correction based on unique molecular tags, and error suppression using normal plasma error profiling incorporated into our new variant caller PlasmaMutationDetector2. In the present study, we assessed the analytical sensitivity and specificity of this method, and demonstrated its usefulness by analyzing plasma samples from patients with advanced pancreatic cancer.

## Results

### Methodological approach

**Figure 1:**
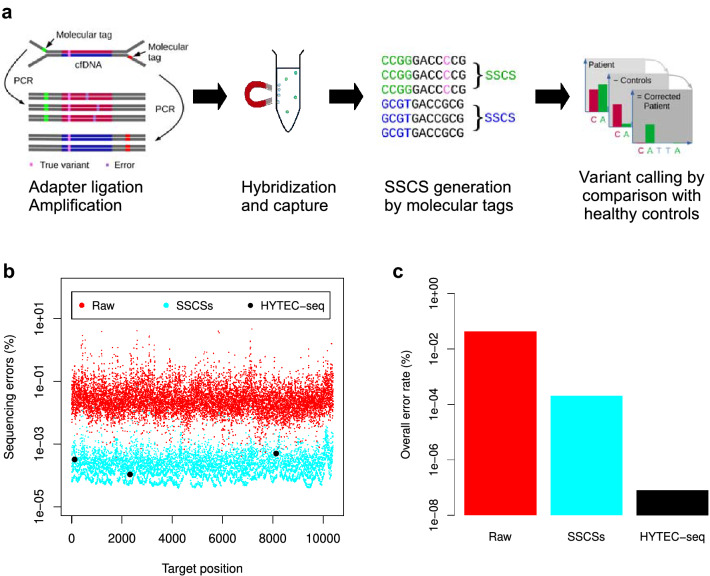
Sequencing error reduction by the HYTEC-seq approach. (**a**) An overview of the HYTEC-seq approach. (**b**) Relative percentage of sequencing errors (substitutions) per target position for raw sequencing reads, single-strand consensus sequences (SSCSs), and the overall HYTEC-seq pipeline (SSCSs + PlasmaMutationDetector2 variant caller). (**c**) Overall error rates for the same three approaches.

To enhance ctDNA detection, we developed a highly sensitive targeted sequencing approach for Ion Torrent chemistry using advanced sequencing error correction (Fig. 1a). We first designed Y-shaped adapters (Supplementary Fig. S1) to improve adapter ligation, and to allow sequencing of both strands in the ligated DNA fragment. This design was intended to reduce the formation of adapter dimers, and the ligation of A–A and P1–P1 adapter pairs on cfDNA fragments. Apart from the Ion Torrent A and P1 adapter sequences, the adapters also contained molecular tags. These tags were used to collapse all sequencing reads of the same cfDNA strand (containing identical molecular tags) into a single-strand consensus sequence (SSCS), and to remove all variants not present in > 70% of the reads having the same tag, as these were presumed to be errors (Supplementary Fig. S2). Subsequently, we filtered the SSCSs for PCR and sequencing errors found in cfDNA samples from individuals without cancer, using a sophisticated statistical algorithm implemented in the new variant caller PlasmaMutationDetector2 (Fig. 1a). In this study, our sequencing approach involved the use of hybridization-based capture to target eight genes that are most frequently mutated in pancreatic cancer (Supplementary Table S1).

### Analytical sensitivity and specificity of HYTEC-seq

To assess the specificity of our new HYTEC-seq technology, we sequenced plasma cfDNA from 60 healthy individuals, and compared the number of errors per sequenced base across the target panel for the various data processing levels (Fig. 1b, c). We found that the relative number of sequencing errors was significantly lower when applying the overall HYTEC-seq bioinformatic pipeline, compared to both raw sequencing reads and after collapsing SSCSs based on molecular tags (*p* < 1E−15). The HYTEC-Seq method led to false detection of three variants (Fig. 1b and Supplementary Table S2), which had variant allele frequencies (VAFs) ranging from 0.24 to 1.82%, representing a final error rate of 8.1E−10 errors/base sequenced, corresponding to specificity rates of > 99.99% at the variant level and 95% (57/60) at the individual level. Further investigation by droplet digital PCR (ddPCR), and comparison with known targets of clonal hematopoiesis of indeterminate potential (CHIP), demonstrated that the three remaining variants were not caused by CHIP, and thus were errors. The falsely positive variant with an allele frequency of 1.8% was supported by only three reads among an overall coverage of 165 reads.

**Figure 2:**
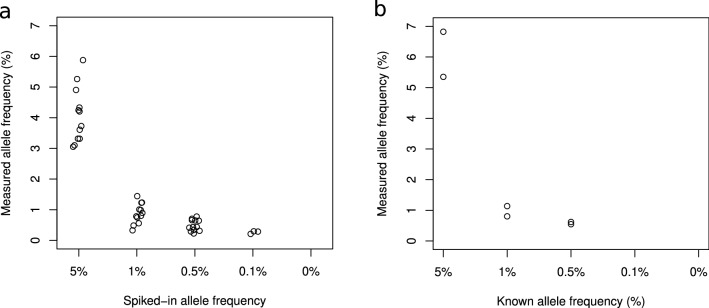
Measured variant allele frequency of known variants detected by the HYTEC-seq method using de novo variant calling. (**a**) Custom-made dilutions of fragmented cell line DNA in fragmented normal leukocyte DNA (50 ng input). (**b**) Multiplex I cfDNA Reference Standard Set (50 ng input). All samples were analyzed in duplicates.

Next, we examined the assay’s analytical sensitivity by analyzing duplicate control samples made by spiking fragmented mutant DNA (~ 160 bp) from four cancer cell lines (AsPC-1, BxPc-3, HCT-116, and HT-29) into fragmented normal leukocyte DNA (~ 160 bp) at known levels (5%, 1%, 0.5%, 0.1%, and negative control) confirmed by ddPCR. These cell lines were chosen because they contained six known mutations in the genes *KRAS*, *SMAD4*, *TP53,* and *RNF43*, enabling us to test our method’s sensitivity across several genes included in the panel. Through application of the PlasmaMutationDetector2 de novo variant caller, we detected all six expected mutations in both duplicates of the 5%, 1%, and 0.5% control samples, and detected 3/12 expected mutations in the 0.1% samples (50 ng input; Fig. 2a). No false positives were called. When using 20 ng input and DNA from identical dilution series, we identified all expected mutations in the 1% and 5% samples, 9/12 expected mutations in the 0.5% samples, and no mutations in the 0.1% samples (Supplementary Fig. S3). However, manual assessment of the SSCS reads (without variant calling) identified all of the expected variants in all spike-in samples when using 50 ng DNA input (except in the negative control samples). Similarly, when using this targeted approach with 20 ng DNA input, we observed all expected mutations in the 5%, 1% and 0.5% spike-in samples, and 10/12 expected mutations in the 0.1% samples. To further validate the method, we analyzed 50-ng aliquots of the commercial Multiplex I cfDNA Reference Standard Set (duplicate samples). Only one of the known mutations in this sample set (*KRAS* G12D) was covered by our gene panel. SSCS reads with the G12D mutation were displayed for all reference samples with known mutations, except in one of the 0.1% samples. De novo variant calling using PlasmaMutationDetector2 consistently detected ctDNA in the 5%, 1%, and 0.5% samples (Fig. 2b). Overall, these results demonstrated that the HYTEC-Seq approach showed sufficient analytical sensitivity to reliably detect variants at allele frequencies as low as 0.1%, which was dependent on the amount of cfDNA input.

We next wanted to benchmark HYTEC-seq against a commercially available ctDNA detection assay available for the Ion Torrent platform. To this end, we analyzed the Multiplex I cfDNA Reference Standard Set using the Oncomine Pan-Cancer Cell-Free Assay (ThermoFisher Scientific) with 50 ng DNA input. In contrast to our results with the HYTEC-seq method, the *KRAS* G12D variant was detected in all mutated control samples (including the 0.1% sample) using the supplier’s variant caller. However, we also observed five false positive variants in the overall panel (Supplementary Fig. S4b). This lower specificity of the Oncomine system was confirmed when we analyzed cfDNA from eight healthy volunteers, which revealed four variants with allele frequencies of ≥ 0.25% that were either false positives or caused by CHIP (Supplementary Table S3).

### Analyzing cfDNA from patients with pancreatic cancer

To demonstrate the performance of the HYTEC-seq method in patient samples, we analyzed cfDNA from plasma samples, obtained from 44 patients with advanced pancreatic cancer before treatment initiation, for the presence of tumor-specific mutations as a surrogate marker for ctDNA. The patients’ median age was 66 years (range 41–81 years), 57% were male (57%), and 91% had stage IV cancers. The median cfDNA input was 20.6 ng (range 2.7–73.4 ng), the median raw sequencing coverage was 67,034×, and the median SSCS coverage was 2234×. The median molecular recovery was 22.1% (range 13.2–33.6%) (Supplementary Fig. S5). Of the sequenced panel positions, only 0.2% had SSCS coverage of < 100 and were not considered for variant calling.

**Figure 3:**
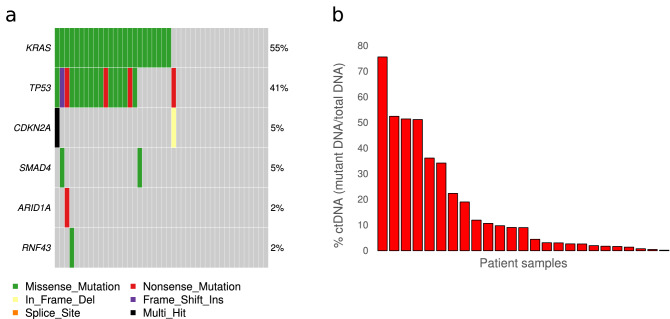
Mutations detected by HYTEC-seq analysis of cfDNA from patient plasma samples. (**a**) Distribution of mutations in patient samples across the target panel. Box color indicates mutation type, as explained in the legend. (**b**) Variant allele frequencies of the mutations in decreasing order. Only the highest allele frequency per sample is shown.

In 31 of the 44 patient samples, PlasmaMutationDetector2 revealed a total of 68 variants (median, 2 variants per sample; range 1–5). To exclude potential false positives, we used ddPCR to validate variants with < 0.5% AF or < 4 SSCSs supporting the variant. We also analyzed DNA from patients’ peripheral blood mononuclear cells to exclude variants caused by clonal hematopoiesis of indeterminate potential (CHIP)^20^. These tests revealed that three variants in *TP53* were due to CHIP, and 12 variants were false positives not confirmed by ddPCR. An additional three variants were excluded due to being synonymous variants. Thus, a total of 25/44 (57%) patients had ctDNA detected based on 50 variants observed in the analyzed samples (median, 2 variants per sample; range 1–4) (Fig. 3, Supplementary Table S4). These included variants in *KRAS* (24 samples), *TP53* (19 samples), *SMAD4* (2 samples), *CDKN2A* (2 samples), *ARID1A* (1 sample), and *RNF43* (1 sample). No mutations were detected in *GNAS* or *TGFBR2*. One patient sample contained two unique *KRAS* variants, while another sample contained two variants in *CDKN2A*. Only one ctDNA-positive sample contained no *KRAS* variants, and instead contained variants in *TP53* and *CDKN2A* (Fig. 3a). The relative ctDNA concentration in the patient samples, determined based on the variant with the highest VAF, ranged from a VAF of 0.23–75.66% (Fig. 3b).

**Figure 4:**
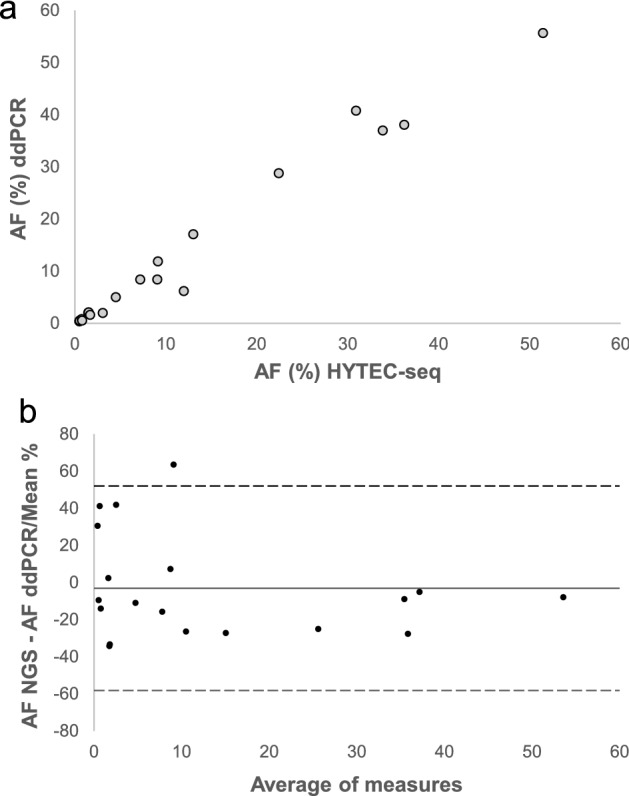
Agreement between HYTEC-seq and droplet digital PCR (ddPCR) results. (**a**) Concordance between variant allele frequency (VAF) measured using HYTEC-seq and ddPCR (Spearman correlation coefficient = 0.964, *p* < 0.001). (**b**) Bland–Altman plot describing the agreement between HYTEC-seq and ddPCR. Solid line indicates bias (mean difference between methods). Dashed lines indicate limit of agreement (± 2 SD).

We further evaluated the concordance in observed VAF between HYTEC-seq and ddPCR by analyzing selected *KRAS* and *TP53* mutations detected by HYTEC-seq in 17 patient samples (VAF range 0.47–51.22%). The results demonstrated that HYTEC-seq and ddPCR were highly concordant (Spearman correlation coefficient = 0.964, *p* < 0.001; Fig. 4a). Only one sample was an outlier, showing a substantial difference in VAF between HYTEC-seq (11.97%) and ddPCR (6.20%; Fig. 4b). This difference might have been caused by a large amount of high-molecular-weight DNA in the sample, likely due to cell lysis during sample handling. This would affect amplicon-based methods, such as ddPCR, but not capture-based methods, in which high-molecular-weight DNA is removed during library construction.

**Figure 5:**
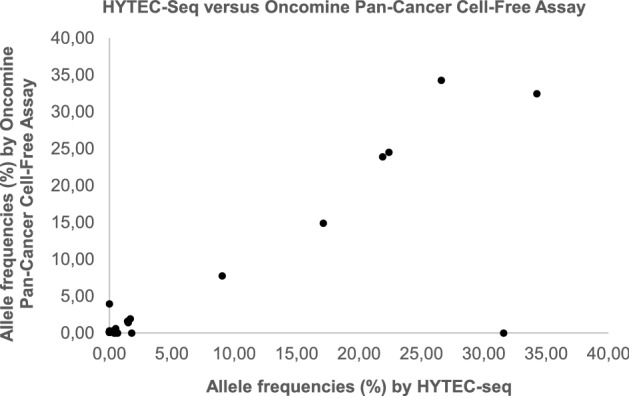
Correlation between HYTEC-seq and Oncomine Pan-Cancer Cell-Free Assay. Comparison of allelic frequencies as determined by HYTEC-seq and Oncomine Pan-Cancer Cell-Free Assay (ThermoFisher Scientific), based on the analysis of 12 patient samples with variable cfDNA input.

Next, we evaluated the performance of our HYTEC-Seq method in patient samples in comparison with the commercially available Oncomine Pan-Cancer Cell-Free Assay (ThermoFisher Scientific). We used both methods to analyze 12 patient samples with variable cfDNA input (range 2.5–50.0 ng). Both methods yielded the identification of 11 variants in target genes that overlapped between the two panels (*KRAS, TP53, SMAD4*, and *GNAS*). In 8/12 patient samples, the variants were detected in *KRAS* (8 samples) and/or *TP53* (3 samples). Additionally, nine variants (VAF range 0.08–3.96%) were detected only with the Oncomine Pan-Cancer Cell-Free Assay, while three variants (VAF range, 0.49–31.60%) were exclusively detected by HYTEC-seq, demonstrating an assay concordance of 48% (11/23). Among the concordant cases, VAF showed a high correlation between the methods (Spearman correlation coefficient = 0.98, *p* < 0.001) (Fig. 5). However, a *TP53* variant with a high VAF (31.60%) detected by HYTEC-seq was not called by the Oncomine Pan-Cancer Cell-Free Assay due to filtering by the associated variant caller.

## Discussion

Digital droplet PCR (ddPCR) is considered the reference method for detection of rare DNA variants due to its high sensitivity^21^, and is thus the method of choice in many ctDNA studies. However, the use of ddPCR analysis to track mutations in plasma requires prior information about the mutations present in the primary tumor or metastases. In contrast, deep sequencing-based methodologies enable high-throughput multigene profiling without prior knowledge of the tumor mutation status, and can potentially detect ctDNA originating from all parts of a tumor in cases of intratumoral heterogeneity. Therefore, here we developed a novel hybridization-based mutation detection method for Ion Torrent chemistry, called HYTEC-seq, in which we correct for PCR and sequencing errors using molecular tags and normal plasma error filtering (Fig. 1a). A similar approach for sequencing error reduction, called integrated digital error suppression (iDES), has previously been reported for Illumina sequencing chemistry^13^. The iDES approach enabled the authors to reduce the error rate to 1E−5 errors per sequenced base in samples from healthy controls, compared to 1E−9 for HYTEC-seq. A more recently described TEC-seq approach also demonstrated a higher error rate (3E−7 errors/base)^22^ than HYTEC-seq. The greater error reduction with HYTEC-seq is largely due to the normal plasma error filtration approach implemented in our new variant caller, PlasmaMutationDetector2, which caused a 1600-fold reduction of errors compared to the 200-fold reduction via molecular tags (Fig. 1b, c). This strong error reduction through normal plasma error profiling has been previously demonstrated, even for sequencing data without molecular tags^14^. The low error rate of HYTEC-seq is also reflected in its high specificity, which was > 99.99% at the variant level and 95% at the sample level, similar to in a prior report^13^.

The HYTEC-seq method demonstrated a high sensitivity with the reproducible de novo detection of variants at a VAF of 0.5%, and even as low as 0.1%, using control samples (Fig. 2). In our analysis of patient samples, we also detected and verified tumor-specific mutations with a VAF as low as 0.23%. Prior reports using iDES technology and 72 ng input DNA have identified specific spiked-in variants with a VAF of < 0.1%, and non-verified plasma ctDNA variants with a VAF as low as 0.13%^13^. Similarly, TEC-seq reproducibly revealed mutations with a VAF of 0.1%^22^, using up to 250 ng input DNA compared to our maximum input of 50 ng. Therefore, both previously described referred approaches seem comparable to HYTEC-seq, although the lower amount of input DNA in our study is a potential limiting factor. Interestingly, the commercialized version of iDES technology, the AVENIO ctDNA liquid biopsy platform (Roche), reportedly detects 0.1% VAF variants with only 50% sensitivity when using 20–40 ng input DNA^23^.

The high sensitivity and specificity of HYTEC-seq was also confirmed when compared with the commercially available Oncomine Pan-Cancer Cell-Free Assay (ThermoFisher Scientific). The Oncomine assay detected the expected *KRAS* mutation even in the 0.1% Multiplex I cfDNA Reference Standard sample (Horizon Discovery), while HYTEC-seq detected the mutation with a VAF as low as 0.5%. However, this difference came at the cost of the specificity, which at the sample level was only 75% with the Oncomine assay in our hands, compared to 95% with HYTEC-seq. In laboratory tests, there is always a trade-off between sensitivity and specificity, but in the context of de novo variant detection we consider high specificity to be more important. One main difference between the Oncomine system and HYTEC-seq is that Oncomine is amplicon-based, whereas HYTEC-seq uses hybridization-based target capture. This likely explains why the Oncomine system seemed to capture a larger fraction of the input cfDNA (58% vs. 22% molecular recovery for HYTEC-seq). However, in contrast to HYTEC-seq, the Oncomine system did not appear to remove false SSCSs caused by sequencing errors in the molecular tag—a phenomenon that frequently causes over-estimation of the molecular recovery of cfDNA fragments in cases with low DNA input amounts (the observed recovery for Oncomine was 29–195%)^24^. Our median molecular recovery rate was similar to the manufacturer’s specified conversion rate of the library construction kit used (Kapa HyperPrep Kit). Notably, the *KRAS* gene had lower capture than the majority of our targets, suggesting that the Multiplex I cfDNA Reference results are not quite representative of the overall performance of HYTEC-seq. Hybridization-based capture methods also remove high-molecular-weight DNA, if present, contributing to lower molecular recovery in such cases.

Another factor that may reduce the sensitivity of mutation detection by HYTEC-seq is the stringency of the variant caller. PlasmaMutationDetector2 is a major revision of the PlasmaMutationDetector variant caller^14^, adapted to sequencing data with molecular tags. The molecular tags substantially reduce the background error rates compared to untagged sequencing data, causing many target positions to be error-free, even when sequencing samples from many healthy individuals. PlasmaMutationDetector2 handles such positions by estimating a rather conservative upper error rate based on the method of Massart^20^. Accordingly, the variant caller performs better when analyzing increasing numbers of normal controls, because the estimated error profile then approaches the real error profile. Furthermore, PlasmaMutationDetector2 applies Bonferroni correction of *p* values with regards to multiple testing, which is one of the more stringent alternatives^25^. Both choices were made with the aim of increasing the method’s specificity, with the drawback of reduced sensitivity. This relationship is emphasized by our observation that all known variants in the 0.1% diluted control samples were present among the sequencing reads, although not called by PlasmaMutationDetector2.

To evaluate the potential clinical utility of our method, we tested the performance of HYTEC-seq on 44 plasma samples from patients with advanced pancreatic cancer. Somatic de novo variant detection was performed in a biopsy-free manner. HYTEC-seq facilitated detection of cfDNA variants in 25/44 (57%) patients, with a VAF as low as 0.23%, with multiple variants (median, 2) detected in the majority of the samples. The detected VAF was also highly concordant with ddPCR (r = 0.964). The results obtained with HYTEC-seq were comparable to previously reported data on ctDNA from patients with metastatic pancreatic cancer, with reported detection rates ranging from 45 to 72% of the examined patients^26–28^. The clinical utility of the HYTEC-seq method would largely depend on the clinical setting, patient stage, and cancer type. However, we believe that the method’s high sensitivity and extraordinary specificity will be sufficient in most settings, as demonstrated by our tumor-agnostic detection of multiple mutations in patients with pancreatic cancer. In many cases, the sensitivity is also constricted by low cfDNA input rather than due to technical limitations, and could thus be improved by increasing cfDNA input.

When comparing the HYTEC-seq and the Oncomine Pan-Cancer Cell-Free Assay for ctDNA detection in patient samples, variants detected by both methods showed a high degree of correlation in terms of VAF (Spearman correlation coefficient = 0.98, *p* < 0.001) (Fig. 5). The only major difference was a high-frequency hotspot TP53 variant (chr17:7577111, VAF = 31.6%) that was filtered out by the Oncomine Variants filter in the Ion Reporter software, probably because it has not been recorded in the Oncomine database (ref https://www.oncomine.org/resource/login.html). Additionally, several variants (mostly with VAF < 1%) were exclusively called by either HYTEC-seq or the Oncomine Pan-Cancer Cell-Free Assay. These discrepancies are reminiscent of observations reported by Stetson and colleagues when comparing four different ctDNA NGS tests with the same 24 plasma samples. They found that assay discordance was mainly due to technical factors^29^. The number of low-frequency variants exclusively called by the Oncomine Pan-Cancer Cell-free Assay (*n* = 9) was higher than those unique for HYTEC-seq (*n* = 3). This fact, combined with the much lower specificity observed for the Oncomine system when analyzing plasma samples from healthy controls, supports speculation that the majority of variants uniquely called with the Oncomine system might be false positives. Some of the low-frequency variants detected by HYTEC-seq were also found to be false positives by ddPCR validation. This emphasizes the importance of error reduction approaches for deep sequencing data, and analytical validation of ctDNA assays prior to their use in a clinical setting.

The commercial deep sequencing-based methods available for ctDNA detection—such as the Avenio ctDNA Expanded panel (Roche), QIAseq Human Comprehensive Cancer panel (Qiagen), Guardant360^®^ (GuardantHealth), and the Oncomine Pan-Cancer Assay (ThermoFisher)—are quite expensive^30,31^. Our HYTEC-seq approach, on the other hand, offers comparable error reduction, sensitivity, and specificity, but at substantially lower costs. This makes HYTEC-seq a good cost-effective alternative to the presently available commercial solutions. Its availability for the Ion Torrent sequencing platform should be of interest to the many diagnostic laboratories that rely on this sequencing chemistry.

In conclusion, we have developed a new sequencing approach for sensitive detection of DNA variants, which reliably detected variants at allele frequencies as low as 0.1%. Using advanced error subtraction methods, this novel HYTEC-seq method achieved error rates and specificities that are comparable to the best available commercial methods in the field. We further demonstrated the utility of this method for ctDNA detection by analyzing plasma samples from a set of patients with inoperable pancreatic cancer. Additional studies are needed to examine the full potential of the HYTEC-seq method through its application in large clinical studies.

## Materials and methods

All methods described were performed in accordance with the relevant guidelines and regulations, and informed consent was obtained from all participants. The project was approved by the regional committee for medical and health research ethics (REC west).

For more details on the material and methods, please see the Supplementary Methods.

### Samples

To validate the HYTEC-Seq method, we sequenced 50-ng control samples from the Multiplex I cfDNA Reference Standard Set (Horizon Discovery). We made a 0.5% control by diluting one part 5% control in nine parts of the negative control from the kit. We also sequenced duplicate 20 ng and 50 ng samples of fragmented leukocyte DNA spiked with 5%, 1%, 0.5%, 0.1%, and 0% (negative control) of fragmented cell line DNA from four cell lines: AsPC-1 (RRID: CVCL_0152), BxPc-3 (RRID: CVCL_0186), HCT-116 (RRID: CVCL_0291), and HT-29 (RRID: CVCL_0320). These cell lines were purchased from the European Collection of Authenticated Cell Cultures (ECACC) and chosen because they reportedly contain mutations in the genes *KRAS* (AsPc-1, HCT-116), *SMAD4* (AsPc-1, HT-29), *TP53* (AsPc-1, BxPc-3), and *RNF43* (AsPc-1). All human cell lines have been authenticated using STR profiling (ECACC) within the last 3 years. To assess the specificity of our new HYTEC-Seq methodology, we sequenced plasma samples from 60 healthy control persons. To demonstrate the clinical utility of the method, we also sequenced 47 plasma samples obtained before and during treatment from 44 patients with inoperable pancreatic cancer. For samples from patients and healthy controls, the cfDNA input in the library preparation reaction varied between 2.7 and 73.4 ng. To compare the performance of our HYTEC-Seq method with the commercially available Oncomine Pan-Cancer Cell-Free Assay (ThermoFisher Scientific), we used both methods to sequence the 50 ng control samples from the Multiplex I cfDNA Reference Standard Set (Horizon Discovery), cfDNA from 12 patients with advanced pancreatic cancer, and eight control samples from healthy individuals without cancer.

### Isolation of circulating cell-free DNA

Peripheral blood was collected from patients and control individuals into EDTA tubes, and subjected to Lymphoprep gradient centrifugation, since we were also interested in isolating mononuclear blood cells for this project. Then the plasma fraction was centrifuged at 17,000×*g* for 10 min to remove cell remnants and remaining platelets. From 4 mL of the cleared plasma fraction, cfDNA was isolated using the QIAamp Circulating Nucleic Acid Kit (Qiagen) following the manufacturer’s protocol. The cfDNA was eluted in 40 µL Buffer AVE, and stored at − 80 ℃ until further use.

### Design and preparation of adapters for HYTEC-seq library construction

The Y-adapters for HYTEC sequencing library construction were designed for sequencing on the Ion Torrent platform. They contained Ion Torrent A and P1 sequences, sample barcode sequences, and molecular tags for error correction. The adapter design is described in detail in the Supplementary Methods (Supplementary Fig. S1). All adapter oligonucleotides were manufactured by IDT DNA technologies with extraordinarily low cross-contamination (TruGrade oligos, max 0.05% cross-contamination) to avoid erroneous de-multiplexing of the sequences. The Y-adapters were constructed by annealing 20 µL of each of the two novel 100 µM P1 and A-adapter oligos in a 50-µL reaction volume, using a modification of a previously published protocol^13^. Briefly, the adapter oligonucleotides were heated to 97.5 °C for 150 s in a thermocycler with a heated lid, and then the machine was turned off and left for 1 h for renaturation of the adapters. Following renaturation, the adapters were diluted in ddH_2_O to appropriate concentrations depending on the cfDNA input.

### Enzymatic fragmentation of leukocyte and cell line DNA

Leukocyte and cell line DNA was enzymatically fragmented using the Ion Xpress™ Plus Fragment Library Kit (ThermoFisher Scientific), following the manufacturer’s instructions, with a target median fragment size of 160 bp. After fragmentation, the samples were purified and size-selected (0.9 × followed by 1.6 × SPRI cleanup) using Agencourt AMPure XP beads (Beckman Coulter). Size was confirmed using the High Sensitivity DNA kit on an Agilent 2100 Bioanalyzer (Agilent Technologies).

### Library preparation for HYTEC-Seq

Sequencing libraries were constructed using the Kapa HyperPrep Kit (Roche), following the manufacturer’s protocol. The libraries were subjected to target capture using “SureSelect Target Enrichment System for Sequencing on Ion Proton” (Agilent Technologies), and a capture panel for eight genes that are frequently mutated in pancreatic cancer. These genes were selected based on previous publications^15–17^ and IGCG data, and included AT-rich interaction domain 1A (*ARID1A*), transforming growth factor beta receptor 2 (*TGFBR2*), cyclin-dependent kinase inhibitor 2A (*CDKN2A*), KRAS proto-oncogene GTPase (*KRAS*), ring finger protein 43 (*RNF43*), tumor protein p53 (*TP53*), SMAD family member 4 (*SMAD4*), and GNAS complex locus (*GNAS*). Probe design is described in the Supplementary Methods. Captured DNA was amplified by PCR, followed by clean-up using Agencourt AMPure XP beads. Then the final DNA libraries were assessed on an Agilent 2100 Bioanalyzer (Agilent Technologies).

### Oncomine Pan-cancer cell-free assay

For the Oncomine Pan-Cancer Cell-Free Assay (ThermoFisher Scientific), libraries were prepared, amplified, and purified according to manufacturer’s protocol. The final library quality and concentration were assessed using the High Sensitivity DNA Assay (Agilent) on an Agilent 2100 Bioanalyzer.

### Ion semiconductor sequencing

Before sequencing, all individual barcoded libraries were diluted to 100 pM. For each run, 4–16 libraries were combined and loaded onto the PI chip for high-depth sequencing on an Ion Proton instrument (Thermo Fisher Scientific) using Ion PI™ Hi-Q™ Sequencing 200 chemistry (Thermo Fisher Scientific).

### Bioinformatic processing of sequencing data

Supplementary Fig. S7 presents an overview of the bioinformatic pipeline for analysis of the HYTEC sequencing data. Signal processing, base calling, quality control, adapter trimming, sample barcode de-multiplexing, and alignment were performed with Ion Torrent Suite Software version 5.12, using the default settings, except for the blind calibration. The sequencing coverage and quality statistics for each sample are summarized in Supplementary Table S7. On-target reads were extracted from aligned BAM files, and the forward and reverse reads were separated and converted to FASTQ format using the samtools software (versions 1.8 and 1.10)^18^. Remaining adapter sequences (duplex part) were removed, and the molecular tag sequence was extracted using a locally developed Python script called TagXtractor (https://github.com/oddmundn/TagXtractor). Subsequently, the reads were aligned to the reference genome using BWA-MEM (version 0.7.17)^19^, producing aligned and sorted BAM files. These BAM files were processed using a Python script called SSCScreator (https://github.com/oddmundn/SSCScreator), version 1.3, which utilized both molecular tag and genome alignment position to collapse reads into single-strand consensus sequences (SSCS). The SSCS included only variants that were present in the majority (> 70%) of reads derived from the same original cfDNA molecule. Subsequently, the forward and reverse SSCS files were combined and aligned to the reference genome, again using BWA-MEM.

For the Oncomine sequencing data, the raw data were first processed using the Ion Torrent Suite Software version 5.12, and were then uploaded to the Ion Reporter Software version 5.10.5.0 (Thermo Fisher Scientific) for variant detection and analysis. This analysis was performed using the Oncomine TagSeq Pan-Cancer Liquid Biopsy w2.1 workflow, and a minimum of two independent molecular families was required to identify a variant for it to be called. Sequencing variants were filtered according to the filter chain Oncomine variants (5.10). For sensitive detection of variants at allele frequencies as low as 0.1%, optimal results are obtained when targeting a median read coverage of > 25,000 and median molecular coverage of > 2,500, and both numbers of the LOD segment are ≤ 0.1%.

### Variant calling and annotation

Aligned single-strand consensus sequence (SSCS) BAMfiles were subjected to variant calling using the PlasmaMutationDetector2 R package version 1.1.10, which we derived from the previously published package PlasmaMutationDetector^14^. A background error profile was constructed using control plasma samples. Then variant calling was performed for patient samples using position-by-position testing for each non-SNP position. Proper multiplicity control was applied to ensure that the global false positive error rate (i.e., the probability of declaring a wild-type sample to be mutated) was smaller than a given user-fixed value. We also required a minimum SSCS coverage of 100 in a variant position, limited strand bias, and the presence of both forward and reverse SSCSs supporting the variant. Only indels longer than 2 bases were considered. Variant annotation was supported by ANNOVAR software version 2018-04-16, and oncoplots were generated using the maftools R package version 2.4.05.

### Digital droplet PCR

To confirm the mutation allele frequencies of the spiked samples from the sensitivity experiments, we analyzed each spiked sample by digital droplet PCR (ddPCR). We used mutation-specific assays for the p.G12D (c.35 C<T) and p.G13D (c. 38 C<T) mutations on the QX200™ Droplet Digital™ PCR system (Bio-Rad), following the manufacturer’s instructions. Mutation analysis was performed with blinding to mutation frequencies, and included appropriate control samples. To validate the variants detected by HYTEC-seq, to detect CHIP variants, and to confirm the sensitivity of our HYTEC-seq method, we performed mutation-specific assays for variants in *KRAS*, *TP53*, and *TGFBR2*, using cfDNA and mononuclear cells as input, respectively. The limit of detection was an allele frequency (VAF) of < 0.1% for single wells using mutation-specific assays.

### Statistical information

The statistical analyses associated with the bioinformatic processing of sequencing data are described above as well as in the Supplementary Methods. Sequencing error rates were compared using the two-tailed Chi-square test. The concordance between detection methods was tested using Spearman’s correlation and Bland–Altman plotting. A *p* value of < 0.05 was considered significant.

### Ethical approval and informed consent

This project was approved by the Regional Committee for Medical and Health Research Ethics (REK-Vest 2011/475), and all participants provided written informed consent.

## Supplementary Information

Supplementary Information 1.

Supplementary Information 2.

## Data Availability

The data that supports the findings of this study are available from the corresponding authors upon reasonable request. The TagXtractor, SSCScreator, and HYTEC-pipeline.sh scripts are available for download at GitHub (https://github.com/). The PlasmaMutationDetector2 R package is available at CRAN (https://cran.r-project.org/).

## Abbreviations

ARID1A: AT-rich interaction domain 1A
CDKN2A: Cyclin-dependent kinase inhibitor 2A
cfDNA: Cell-free DNA
CHIP: Clonal hematopoiesis of indeterminate potential
ctDNA: Circulating tumor DNA
ddPCR: Droplet digital PCR
GNAS: GNAS complex locus
HYTEC-seq: HYbridization- and Tag-based Error-Corrected sequencing
iDES: Integrated digital error suppression
KRAS: Kirsten RAS proto-oncogene GTPase
LOD: Limit of detection
RNF43: Ring finger protein 43
SMAD4: SMAD family member 4
SSCS: Single-strand consensus sequence
TGFBR2: Transforming growth factor beta receptor 2
TP53: Tumor protein p53
UMI: Unique molecular identifier
VAF: Variant allel frequency
