# Supplementary material for: Novel hybridization- and tag-based error-corrected method for sensitive ctDNA mutation detection using ion semiconductor sequencing
Source: Sci Rep. 2022 Apr 6;12:5816. doi: 10.1038/s41598-022-09698-5 (PMC8986848; doi:10.1038/s41598-022-09698-5)
Supplement: Supplementary file 1 — Supplementary Information 1. [file 41598_2022_9698_MOESM1_ESM.docx]

**Supplementary information file**

**Novel hybridization- and tag-based error-corrected method for sensitive ctDNA mutation detection using ion semiconductor sequencing**

Kjersti Tjensvoll*^1^, Morten Lapin^1^, Bjørnar Gilje^1^, Herish Garresori^1^, Satu Oltedal^1^, Rakel Brendsdal Forthun^3,4^, Anders Molven^5,6^, Yves Rozenholc^2^ and Oddmund Nordgård^1^

^1^Department of Haematology and Oncology, Stavanger University Hospital, N-4011 Stavanger, Norway.

^2^BioSTM UR 7537, Faculté de Pharmacie de Paris, Université de Paris, 75006 Paris, France.

^3^[Department of](https://www.uib.no/en/clin2) Medical Genetics, Haukeland University Hospital, N-5020 Bergen.

^4^Department of Internal Medicine, Hematology Section, Haukeland University Hospital, N-5020 Bergen, Norway.

^5^Gade Laboratory for Pathology, Department of Clinical Medicine, University of Bergen, N-5020 Bergen, Norway

^6^[Department of](https://www.uib.no/en/clin2) Pathology, Haukeland University Hospital, N-5021 Bergen, Norway

# SUPPLEMENTAL INFORMATION


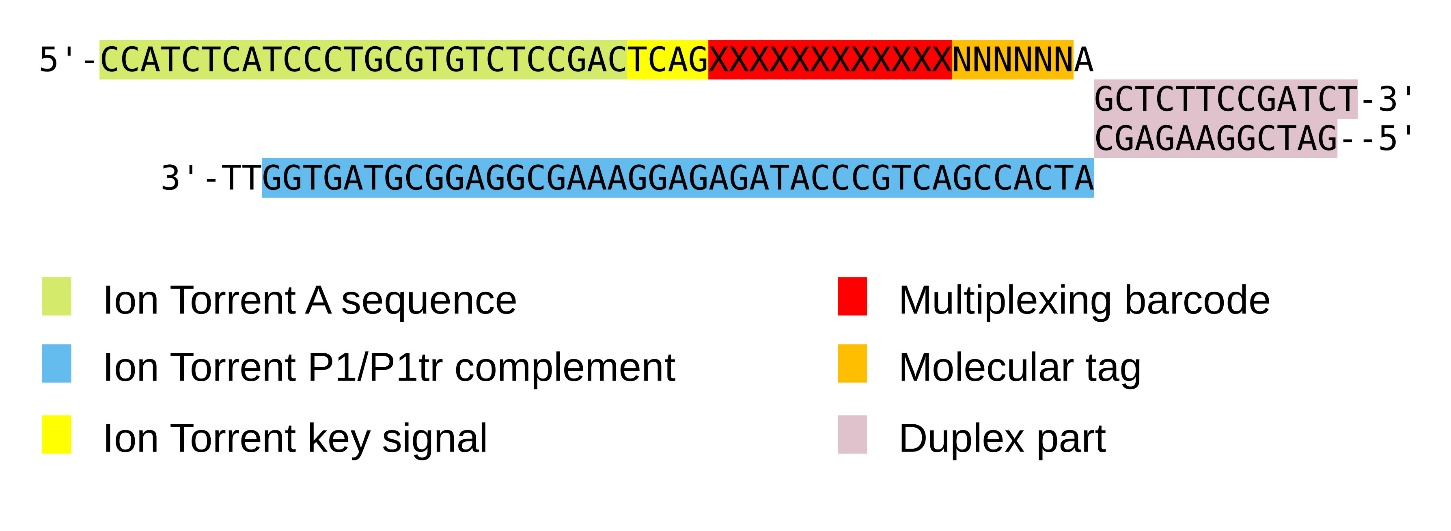


**Supplemental Figure 1:** Adapter design. The figure shows the adapter sequences and the structural elements are indicated by coloured boxes.


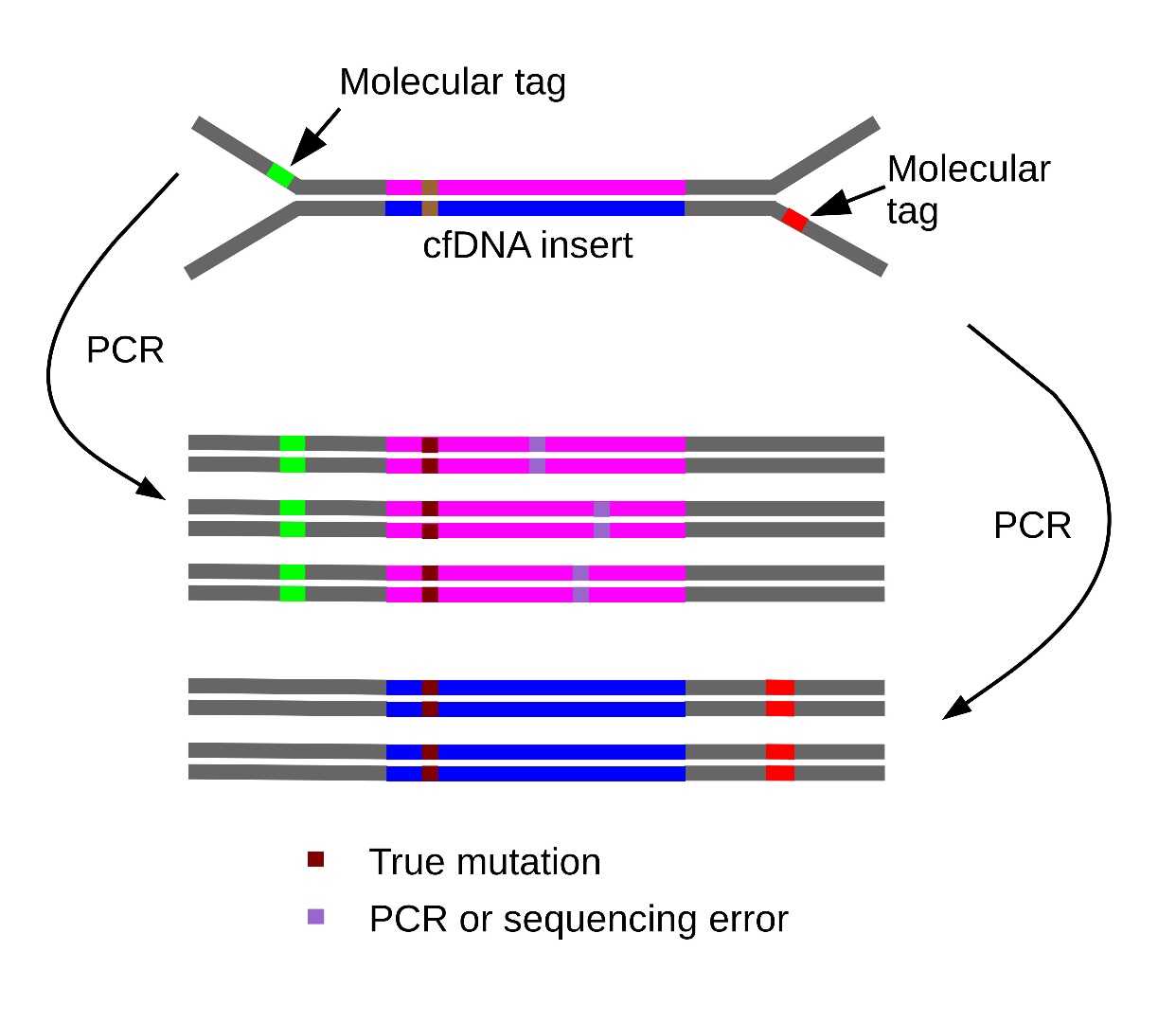


**Supplemental Figure 2:** Principle of how molecular tags in the sequencing adapters are utilized to remove sequencing errors. Only variants present in all copies/reads from the same original cfDNA molecule will be accepted.


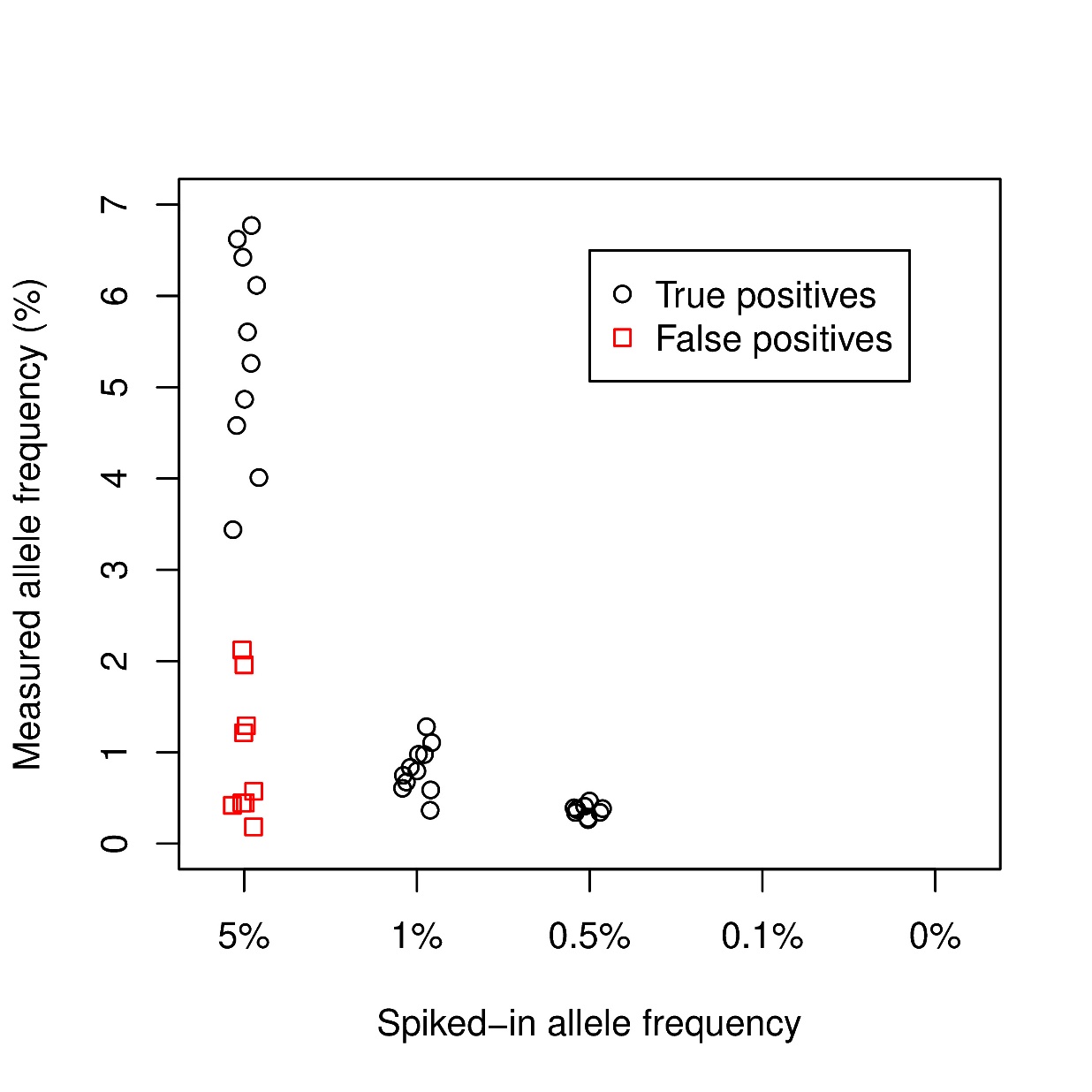


**Supplemental Figure 3:** Measured allele frequencies of known mutations in spiking experiment using 20 ng input DNA. The figure shows known and measured allele frequencies of variants detected by de-novo variant calling in two dilutions series of a cell line mix with 6 known mutations in the HYTEC-seq gene panel. False positives are shown by red squares.


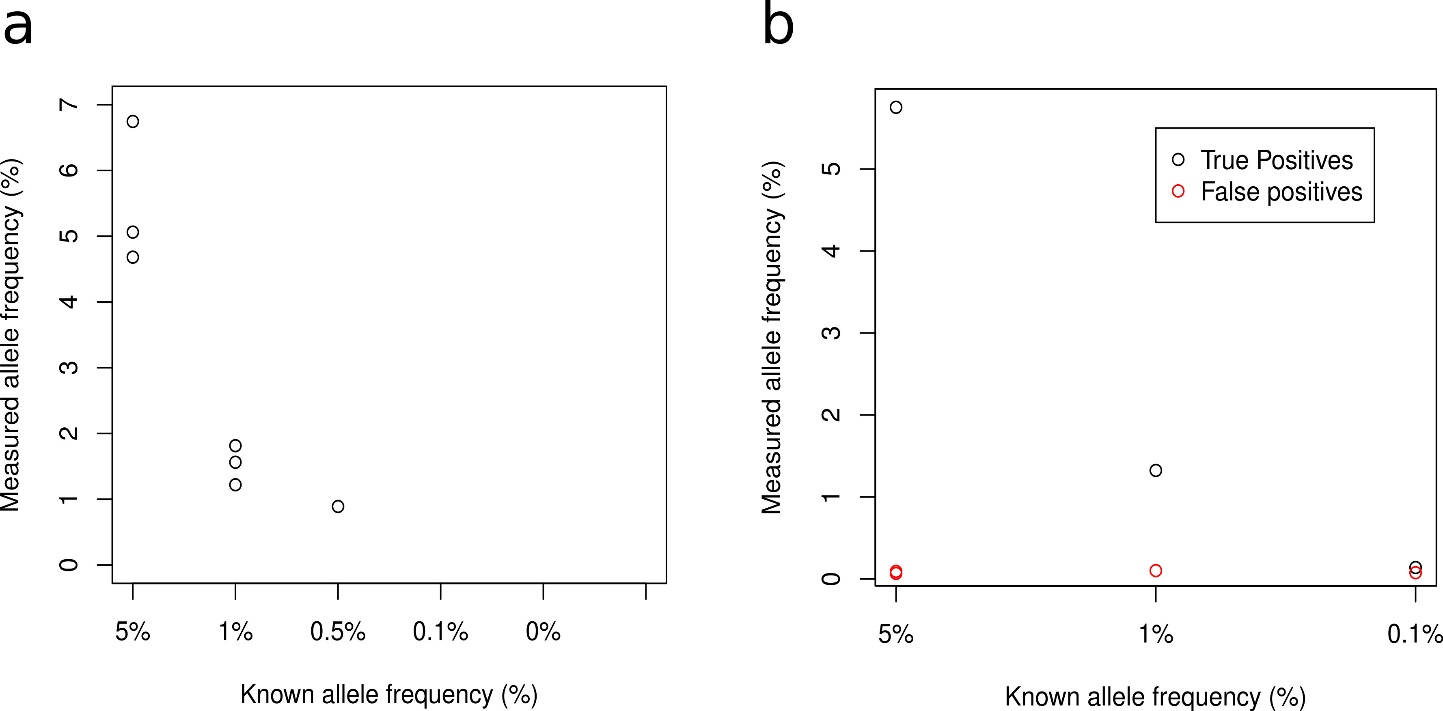


**Supplemental Figure 4:** Measured allele frequencies of *KRAS* mutations detected by HYTEC-seq (a) and Oncomine Pan-Cancer Cell-Free Assay (b) in commercial controls samples. Red circles indicate false positive in the gene panel analyzed. HYTEC-seq analysis was done in triplicates using 20 ng input DNA, Oncomine in single reactions using 50 ng input DNA.

**
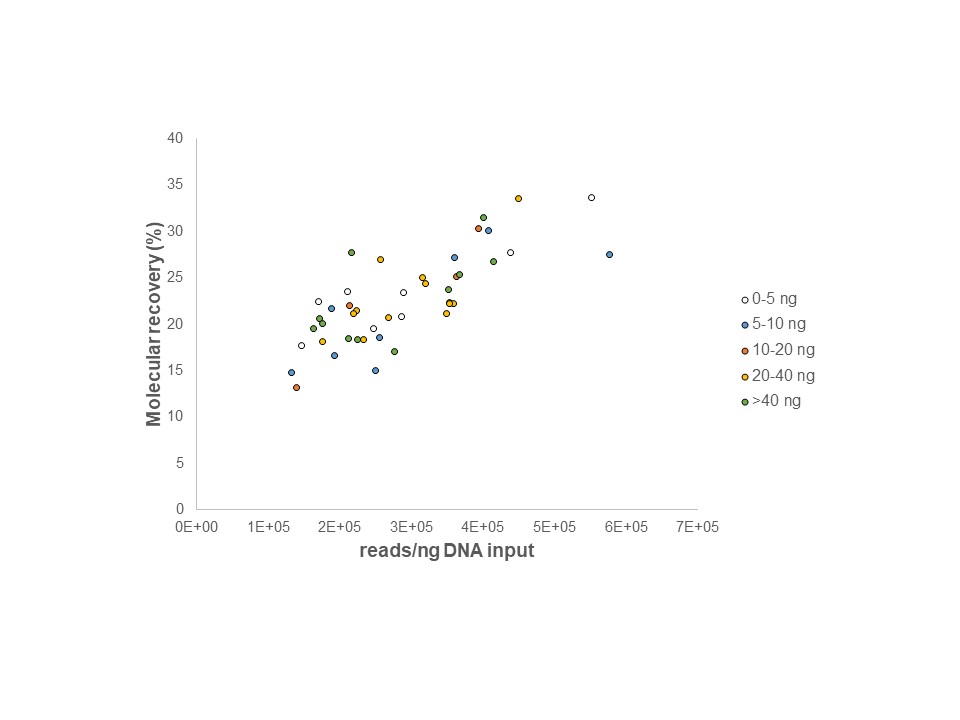
**

**Supplemental Figure 5:** Molecular recovery of input DNA compared to the number of on-target reads per ng DNA input.

**
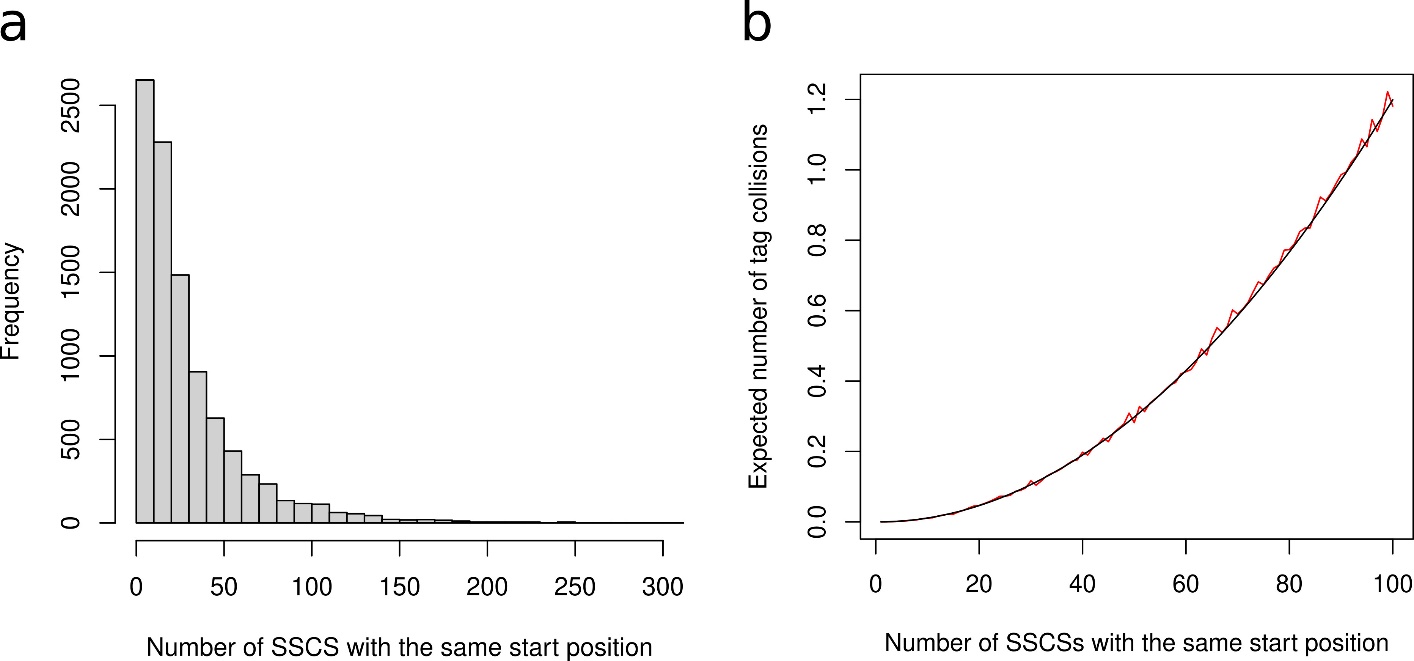
**

**Supplemental Figure 6:** Distribution and expected number of tag collisions for increasing numbers of SSCS with the same starting position. **a** Histogram showing the number of SSCS with the same orientation and starting position for a 50 ng input cfDNA sample. **b** Expected number of tag collisions as a function of the number of SSCS with the same staring position. The black curve shows theoretical estimates and the red curve results from simulations with 10 000 iterations.


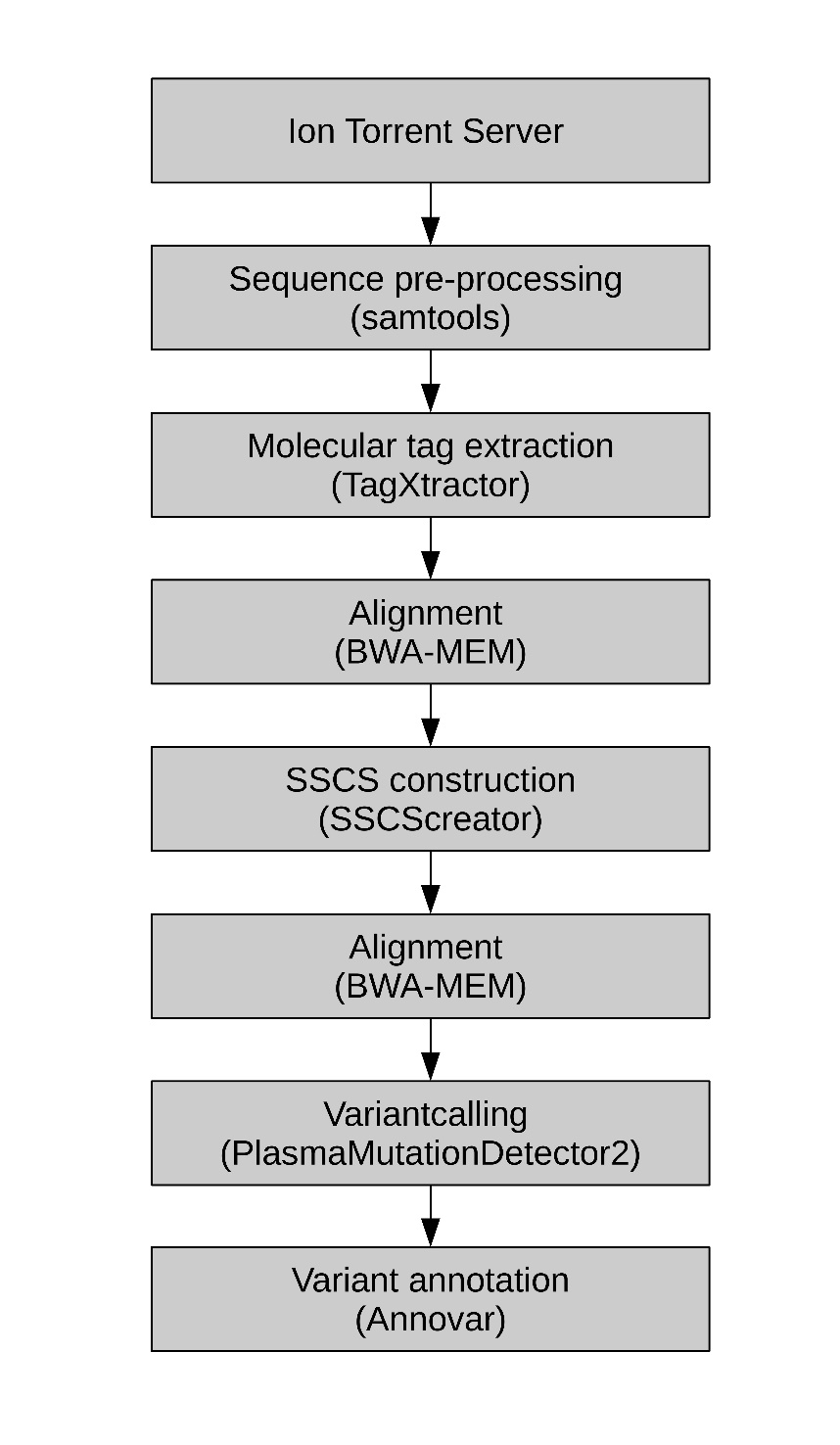


**Supplemental Figure 7**: Bioinformatic pipeline overview

**
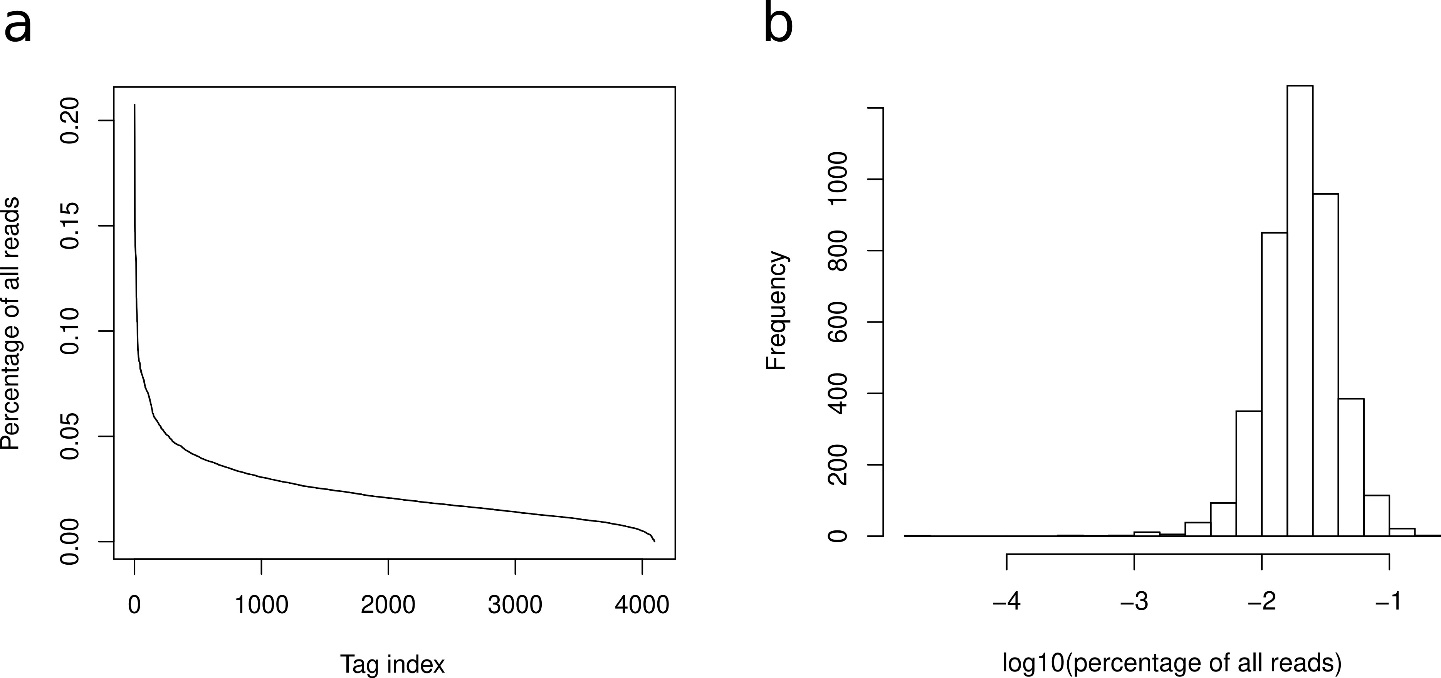
**

**Supplemental Figure 8:** Distribution of tags in a selected sequencing analysis. **a** All tags' representation (%) among all reads, in order from highest to lowest. **b** Histogram of tag representation (%) in logarithmic scale.

##

**
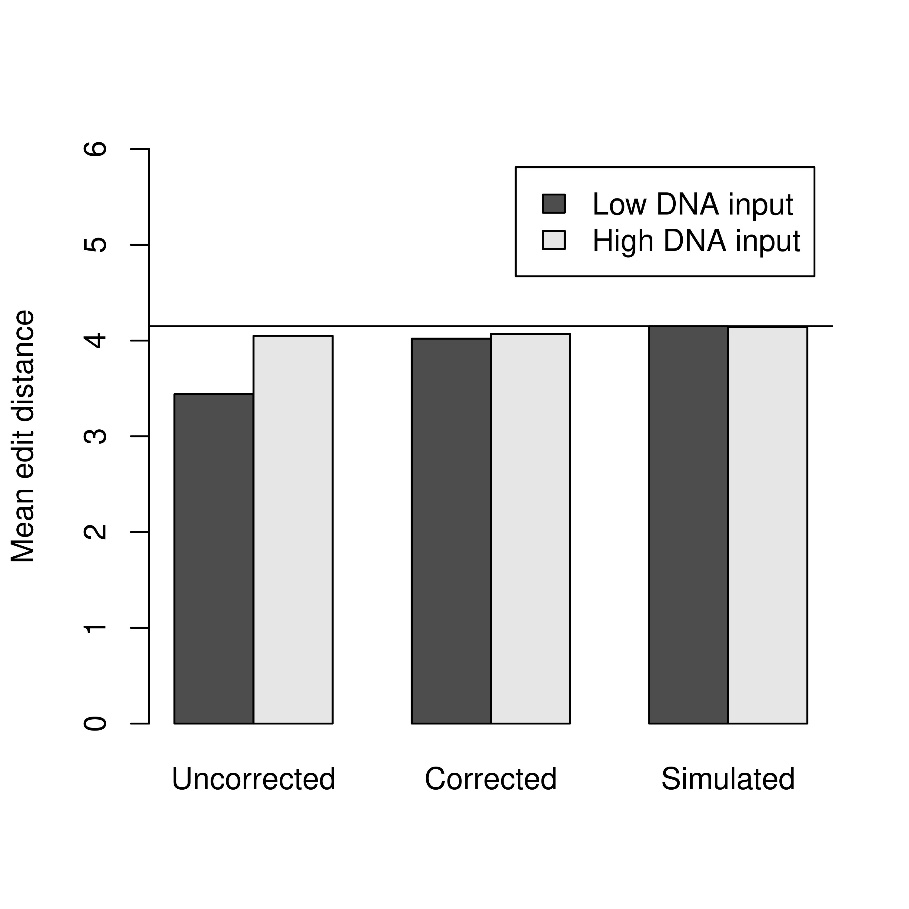
**

**Supplemental Figure 9:** Median edit distance for target positions in which two or more forward SSCS are starting, before and after correction for sequencing errors in molecular tag. Simulated edit distances for randomly selected barcodes are shown for comparison. Data for a sample with little DNA input is shown by black bars, whereas a sample with high DNA input is shown by white bars. The horizontal dotted line shows the edit distance 4.15, which the simulations converge towards.


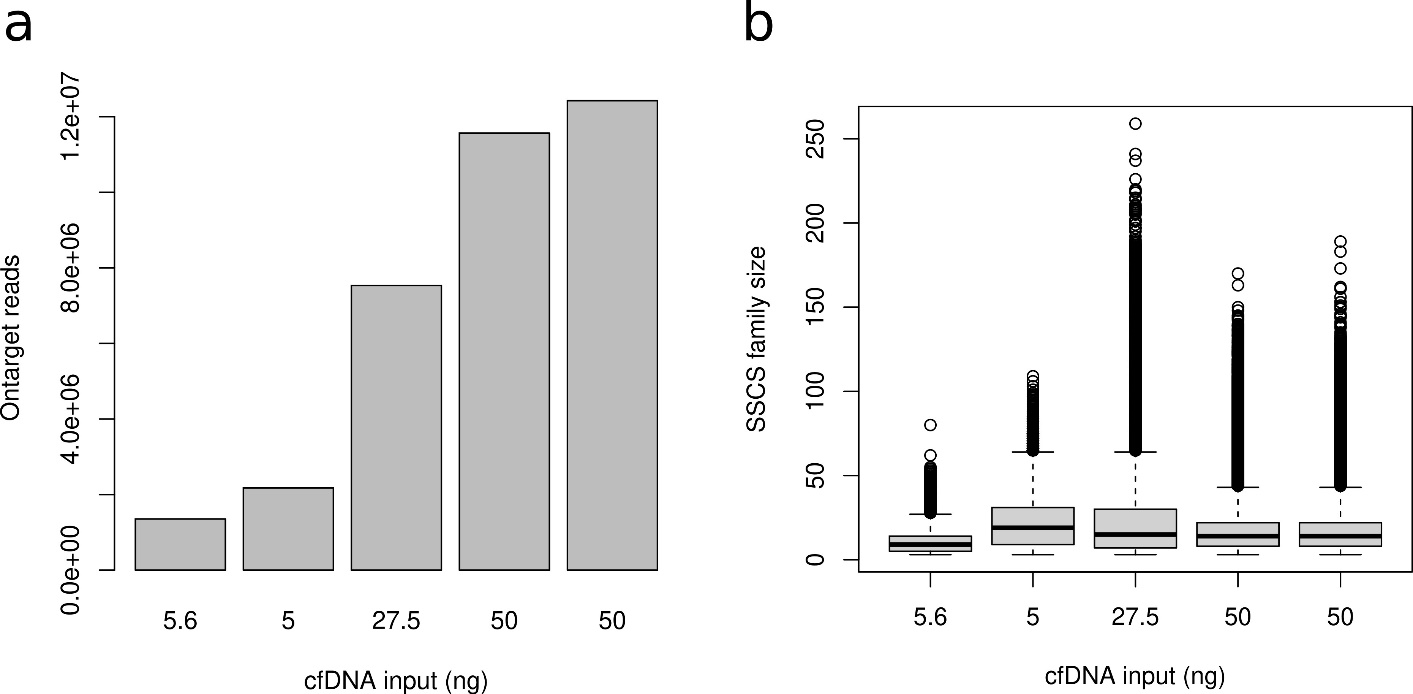


**Supplemental Figure 10: SSCS** family size evaluations. **a** The number of on-target reads for five randomly selected patient cfDNA samples, arranged in increasing order. The amount of cfDNA input is shown below each bar. **b** Boxplot showing SSCS family sizes for the same samples as in A), using the same order of samples.

**Supplemental** [**Table 1:** Target gene panel.](#z337ya)

| **Chr** | **Gene** | **Exons** | **Coverage** | **Length (bp)** |
| --- | --- | --- | --- | --- |
| 1 | *ARID1A* | 2-8, 11-18, 20 | Most coding exons | 4470 |
| 3 | *TGFBR2* | 5-8 | 4/8 coding exons | 850 |
| 9 | *CDKN2A* | 1-3 | All coding exons | 471 |
| 12 | *KRAS* | 2-3 | Hot spots | 290 |
| 17 | *TP53* | 2-11 | All coding exons | 1182 |
| 17 | *RNF43* | 2-9 | Most coding exons | 1499 |
| 18 | *SMAD4* | 2-12 | All coding exons | 1659 |
| 20 | *GNAS* | 9 | Hot spot | 74 |

**Supplemental Table 2:** False positive variants called in iterated analysis of normal plasma samples using our HYTEC-seq panel

| **Sample** | **Genomic-pos.** | **Gene** | **Ref** | **Mut** | **Cov.** | **Allelic-freq (%)** | **Reads +** | **Reads -** | **P-**  **value** |
| --- | --- | --- | --- | --- | --- | --- | --- | --- | --- |
| C50 | chr1:27100122 | ARID1A | G | T | 165 | 1.82 | 2 | 1 | 2.3E-03 |
| C44 | chr18:48584593 | SMAD4 | C | G | 830 | 0.24 | 1 | 1 | 9.4E-03 |
| C56 | chr1:27056325 | ARID1A | G | A | 1079 | 0.56 | 5 | 1 | 4.3E-04 |

**Supplemental Table 3:** False positive variants called in the analysis of normal plasma samples using the Oncomine Pan-Cancer Cell-Free Assay

| **Sample** | **Genomic-pos.** | **Gene** | **Ref** | **Mut** | **Mol. cov.** | **Allelic-freq (%)** | **Mol count mutant** |
| --- | --- | --- | --- | --- | --- | --- | --- |
| C7 | chr17:7578470 | TP53 | T | C | 1639 | 0.16 | 4 |
|  | chr17:7579440 | TP53 | CT | C | 1639 | 0.07 | 1 |
|  | chr17:7579874 | TP53 | AG | A | 1639 | 0.13 | 3 |
| C32 | chr17:7579418 | TP53 | GG | GAG | 1139 | 0.55 | 4 |

**Supplemental Table 4:** Patient characteristics and pretreatment ctDNA results in ctDNA positive patients.

| Sample ID | Age | Sex | Stage | Tumor size (mm) | Tumor location | Gene | Mutation effect | Mutation type | ctDNA (%) | Copies/mL plasma |
| --- | --- | --- | --- | --- | --- | --- | --- | --- | --- | --- |
| P3-1 | 55 | M | IV | 26 | Tail | *TP53* | M237I | SNV | 34,26 | 33298 |
|  |  |  |  |  |  | *KRAS* | Q61H | SNV | 26,60 | 25853 |
|  |  |  |  |  |  | *CDKN2A* | L117del | INDEL | 17,24 | 16760 |
|  |  |  |  |  |  | *CDKN2A* | Splice Site | SNV | 13,02 | 12659 |
| P4-1 | 74 | F | IV | 30 | Head | *KRAS* | G12D | SNV | 22,40 | 898 |
|  |  |  |  |  |  | *TP53* | G245S | SNV | 17,14 | 688 |
| P5-1 | 67 | M | IV | 11 | Head | *KRAS* | G12V | SNV | 0,50 | 14 |
|  |  |  |  |  |  | *TP53* | H179R | SNV | 0,49 | 14 |
| P6-1 | 53 | M | IV | 35 | Tail | *KRAS* | G12D | SNV | 36,21 | 30667 |
|  |  |  |  |  |  | *TP53* | C242Y | SNV | 30,89 | 26161 |
| P8-1 | 79 | M | IV | 63 | Tail | *KRAS* | G12D | SNV | 4,48 | 60 |
|  |  |  |  |  |  | *TP53* | G245S | SNV | 3,20 | 43 |
| P9-1 | 51 | F | IV | 51 | Multiple | *KRAS* | G12D | SNV | 9,12 | 1224 |
| P13-1 | 66 | M | IV | 37 | Head | *KRAS* | G12D | SNV | 9,04 | 250 |
| P15-1 | 69 | M | IV | 60 | Tail | *SMAD4* | W99C | SNV | 9,76 | 1004 |
|  |  |  |  |  |  | *KRAS* | G12V | SNV | 7,17 | 737 |
|  |  |  |  |  |  | *TP53* | H233Gfs*13 | INDEL | 3,01 | 310 |
| P16-1 | 72 | M | IV | 54 | Tail | *KRAS* | G12R | SNV | 10,67 | 124 |
|  |  |  |  |  |  | *TP53* | Y234C | SNV | 8,77 | 102 |
| P19-1 | 50 | M | IV | 68 | Tail | *TP53* | A276D | SNV | 19,05 | 5015 |
|  |  |  |  |  |  | *KRAS* | G12D | SNV | 13,01 | 3424 |
| P22-1 | 58 | F | IV | 30 | Head | *KRAS* | G12V | SNV | 2,61 | 14 |
| P23-1 | 57 | M | IV | 100 | Multiple | *KRAS* | G12D | SNV | 11,97 | 538 |
|  |  |  |  |  |  | *TP53* | E198* | SNV | 9,95 | 447 |
|  |  |  |  |  |  | *ARID1A* | R1461* | SNV | 8,05 | 362 |
| P24-1 | 71 | F | IV | 36 | Multiple | *TP53* | R175H | SNV | 1,67 | 58 |
|  |  |  |  |  |  | *KRAS* | G12D | SNV | 1,50 | 52 |
| P25-1 | 63 | F | IV | 32 | Body | *RNF43* | A146D | SNV | 1,40 | 526 |
|  |  |  |  |  |  | *KRAS* | G12R | SNV | 0,81 | 306 |
|  |  |  |  |  |  | *TP53* | R267G | SNV | 0,68 | 256 |
| P28-1 | 70 | M | IV | 34 | Tail | *SMAD4* | D355Y | SNV | 1,78 | 58 |
|  |  |  |  |  |  | *KRAS* | G12V | SNV | 1,45 | 48 |
| P33-1 | 71 | M | IV | 64 | Head | *KRAS* | G12V | SNV | 75,66 | 2367 |
| P34-1 | 41 | M | IV | 37 | Tail | *KRAS* | G12D | SNV | 3,06 | 15 |
| P36-1 | 44 | F | III | 42 | Head | *KRAS* | G12R | SNV | 2,71 | 29 |
|  |  |  |  |  |  | *TP53* | R196* | SNV | 1,42 | 15 |
| P37-1 | 81 | F | IV | 31 | Head | *TP53* | R196* | SNV | 51,22 | 4917 |
|  |  |  |  |  |  | *KRAS* | G12D | SNV | 30,90 | 2966 |
| P38-1 | 79 | M | IV | 53 | Tail | *KRAS* | G12C | SNV | 3,13 | 920 |
|  |  |  |  |  |  | *TP53* | R267W | SNV | 2,57 | 756 |
|  |  |  |  |  |  | *KRAS* | G12V | SNV | 0,73 | 214 |
| P40-1 | 54 | M | IV | 32 | Head | *KRAS* | G12D | SNV | 0,79 | 31 |
|  |  |  |  |  |  | *TP53* | V173M | SNV | 0,54 | 21 |
| P41-1 | 66 | M | IV | 20 | Tail | *TP53* | S183* | SNV | 1,97 | 15 |
|  |  |  |  |  |  | *CDKN2A* | S43_Y44delinsN | INDEL | 0,78 | 6 |
| P43-1 | 62 | M | IV | 33 | Head | *KRAS* | G12V | SNV | 51,46 | 12566 |
|  |  |  |  |  |  | *TP53* | R273H | SNV | 35,21 | 8598 |
| P44-1 | 65 | M | IV | 50 | Head | *KRAS* | G12V | SNV | 0,23 | 13 |
| P48-1 | 69 | M | IV | 35 | Body | *TP53* | G266V | SNV | 52,50 | 38582 |
|  |  |  |  |  |  | *KRAS* | G12D | SNV | 33,85 | 24877 |

**Supplemental Table 5:** Adapter oligonucleotides for library preparation using our HYTEC-Seq procedure. Sample barcode sequences are underlined

| Name | Sequence | Modifications |
| --- | --- | --- |
| HYTEC-adpt-X1 | 5`-CCATCTCATCCCTGCGTGTCTCCGACTCAG CTAAGGTAACGATNNNNNNAGCTCTTCCGATct-3` | 3’ PTO |
| HYTEC-adpt-X2 | 5`-CCATCTCATCCCTGCGTGTCTCCGACTCAG TAAGGAGAACGATNNNNNNAGCTCTTCCGATct-3` | 3’ PTO |
| HYTEC-adpt-X3 | 5`-CCATCTCATCCCTGCGTGTCTCCGACTCAG AAGAGGATTCGATNNNNNNAGCTCTTCCGATct-3` | 3’ PTO |
| HYTEC-adpt-X4 | 5`-CCATCTCATCCCTGCGTGTCTCCGACTCAG TACCAAGATCGATNNNNNNAGCTCTTCCGATct-3` | 3’ PTO |
| HYTEC-adpt-X5 | 5`-CCATCTCATCCCTGCGTGTCTCCGACTCAG CAGAAGGAACGATNNNNNNAGCTCTTCCGATct-3` | 3’ PTO |
| HYTEC-adpt-X6 | 5`-CCATCTCATCCCTGCGTGTCTCCGACTCAG CTGCAAGTTCGATNNNNNNAGCTCTTCCGATct-3` | 3’ PTO |
| HYTEC-adpt-X9 | 5`-CCATCTCATCCCTGCGTGTCTCCGACTCAG TGAGCGGAACGATNNNNNNAGCTCTTCCGATct-3` | 3’ PTO |
| HYTEC-adpt-X11 | 5`-CCATCTCATCCCTGCGTGTCTCCGACTCAG TCCTCGAATCGATNNNNNNAGCTCTTCCGATct-3` | 3’ PTO |
| HYTEC-adpt-X12 | 5`-CCATCTCATCCCTGCGTGTCTCCGACTCAG TAGGTGGTTCGATNNNNNNAGCTCTTCCGATct-3` | 3’ PTO |
| HYTEC-adpt-X13 | 5`-CCATCTCATCCCTGCGTGTCTCCGACTCAG TCTAACGGACGATNNNNNNAGCTCTTCCGATct-3` | 3’ PTO |
| HYTEC-adpt-X14 | 5'-CCATCTCATCCCTGCGTGTCTCCGACTCAG TTGGAGTGTCGATNNNNNNAGCTCTTCCGATct - 3' | 3’ PTO |
| HYTEC-adpt-X15 | 5'-CCATCTCATCCCTGCGTGTCTCCGACTCAG TCTAGAGGTCGATNNNNNNAGCTCTTCCGATct - 3' | 3’ PTO |
| HYTEC-adpt-X16 | 5'-CCATCTCATCCCTGCGTGTCTCCGACTCAG TCTGGATGACGATNNNNNNAGCTCTTCCGATct - 3' | 3’ PTO |
| HYTEC-adpt-X17 | 5'-CCATCTCATCCCTGCGTGTCTCCGACTCAG TCTATTCGTCGATNNNNNNAGCTCTTCCGATct - 3' | 3’ PTO |
| HYTEC-adpt-X20 | 5'-CCATCTCATCCCTGCGTGTCTCCGACTCAG CAGATCCATCGATNNNNNNAGCTCTTCCGATct - 3' | 3’ PTO |
| HYTEC-adpt-X21 | 5'-CCATCTCATCCCTGCGTGTCTCCGACTCAG TCGCAATTACGATNNNNNNAGCTCTTCCGATct - 3' | 3’ PTO |
| HYTEC-adpt-P1-1 | 5’-GATCGGAAGAGCATCACCGACTGCCCATA GAGAGGAAAGCGGAGGCGTAGTGGTT-3 | 5’ Phosphate +3’ PTO |

##

**Supplemental** [**Table 6**: Additional *CDKN2A* probes](#1y810tw)

| **Probe ID** | **Sequence** |
| --- | --- |
| BA_107514_000079 | GGTTACTGCCTCTGGTGCCCCCCGCAGCCGCGCGCAGGTACCGTGCGACATCGCGATGGCCCAGCTCCTCAGCCAGGTCCACGGGCAGACGGCCCCAGGCATCGCGCACGTCCAGCCGCG |
| BA_107514_000080 | CCAGCTCCTCAGCCAGGTCCACGGGCAGACGGCCCCAGGCATCGCGCACGTCCAGCCGCGCCCCGGCCCGGTGCAGCACCACCAGCGTGTCCAGGAAGCCCTCCCGGGCAGCGTCGTGCA |
| BA_107514_000081 | CCCCGGCCCGGTGCAGCACCACCAGCGTGTCCAGGAAGCCCTCCCGGGCAGCGTCGTGCACGGGTCGGGTGAGAGTGGCGGGGTCGGCGCAGTTGGGCTCCGCGCCGTGGAGCAGCAGCA |
| BA_107514_000082 | CGGGTCGGGTGAGAGTGGCGGGGTCGGCGCAGTTGGGCTCCGCGCCGTGGAGCAGCAGCAGCTCCGCCACTCGGGCGCTGCCCATCATCATGACCTGCCAGAGAGAACAGAATGGTCAGA |

##

## Design of adapters for library construction

The adapter for construction of HYTEC sequencing libraries was designed to have two different single-stranded ends (Y adapters) to enable ligation with the same adapter in both ends of the DNA inserts (Supplemental Figure 1). The adapters were also constructed to include sequences complementary to Ion Torrent sequencing primers to enable sequencing with Ion Torrent chemistry. In addition, IonXpress barcode sequences (Supplemental Table 5) were included for sample multiplexing. A random sequence of 6 bases also provided unique molecular tags for advanced error correction, as described below (Supplemental Figure 2). The duplex part of the sequences were obtained from a previous publication^1^.

The adapter oligonucleotides were manufactured by IDT DNA technologies with extraordinary low cross-contamination (TruGrade oligos, max 0.05% cross-contamination) to avoid erroneous de-multiplexing of sequences. The sequence of the adapter oligonucleotides are shown in Supplemental Table 5.

The length of the molecular tag (6 random bases), combined with unique fragment start positions and orientation, were considered to provide enough uniqueness for de-multiplexing of unique template molecules. In detail, we reasoned that with a mean fragment length of 166 bp a total amount of 50 ng cfDNA will contain around (5.0E-8 g /660 g/mol) x 6,022E23 = 4.6E13 dsDNA nucleotides and 4.6E13/166 = 2,7E11 cfDNA fragments. If we assume that these fragments can start in any position in the single-stranded genome and that we capture 25% of the cfDNA fragments, there will be an expected average of 2.7E11/3E9 x 0.25 = 23 fragments starting in every position. However, we also determined the numbers of SSCS with the same orientation and starting position experimentally based on a typical analysis of 50 ng cfDNA (Supplemental Figure 6a). The observed mean number was 29.9 and the median 20, fitting well with the theoretical estimate. Yet, the data distribution was quite wide and left-skewed, with 5 and 95 percent quantiles at 2 and 93, respectively. As expected, the number of SSCS with the same starting position was lower when less input DNA was analyzed (results not shown).

The expected number of molecular tag collisions (two or more identical tags) were estimated for increasing numbers of SSCSs, using both direct calculations and simulations. The direct calculations were done with the expression

*ExpectedCollisions = n-k+k*(((k-1)/k)^n)*

where n is the number of SSCSs with the same starting position and k is the number of possible tags (4096)^2^. The simulations were done in Python3 (version 3.8.3) and consisted of 10 000 iterations for every input value of k. Supplementary Figure 6B demonstrates that the expected number of tag collisions ranged up to 1.2 for n values up to 100, comprising up to 1.2% of the SSCS number. Thus, the number of tag collisions is well below 1% of the total SSCS number, because the amount of input DNA was never higher than 50 ng (corresponding to 23 fragments with the same start position in average), and we considered the uniqueness for cfDNA fragment labeling to be sufficient.

## Enzymatic fragmentation of DNA for constructions of positive control samples

Using the Ion Xpress™ Plus Fragment Library Kit (Thermo Fisher) 1 µg of leukocyte or cell line DNA was mixed with 5 µL Ion Shear Plus 10x Reaction Buffer (Thermo Fisher) and diluted to 40 µL with nuclease-free water. Ten µL Ion Shear Plus Enzyme Mix (Thermo Fisher) were added, and the sample incubated at 37℃ for 45 min to produce DNA fragments approximately 160 bp in size. After the incubation, 5 µL of Ion Shear Plus Stop Buffer were added, and the samples were purified and size-selected (0.9× followed by 1.6×SPRI cleanup) using Agencourt AMPure XP beads (Beckman Coulter).

## Library preparation

### Adaptor ligation, post-ligation cleanup and library amplification

The Y-adapters were constructed by annealing 20 µL of each of the two novel 100 µM P1 and A-adapter oligos in a 50-µL reaction volume. This was performed by heating the adapter oligonucleotides to 97.5°C for 150 s in a thermocycler (with a heated lid) before the machine was turned off and left for 1 hour for renaturation of the adapters ^3^. After the renaturation, the adapters were diluted in ddH_2_O to appropriate concentrations depending on the cfDNA input.

DNA libraries were constructed from both fragmented control samples, and cfDNA from healthy control and patient samples using the Kapa HyperPrep Kit (Roche). In brief, 0.25 to 73.35 ng fragmented genomic DNA or cfDNA was added to the end-repair and A-tailing reagents and incubated at 20°C for 30 min followed by 65°C for 30 min and cooling at 4°C. Adapters, diluted according to the manufacturer’s recommendation, were then added (one unique Y-adapter for each sample) to the ligation mixture for a total volume of 110 µL. The samples were incubated overnight (16-18 hours) at 4°C for adapter ligation. Samples were directly processed next day with a post-ligation 0.8×SPRI cleanup followed by library amplification using the specified reaction mixture: 30 μL 2× KAPA HiFi Hotstart Ready Mix, 6.0 μL 20 µM primer mix consisting of custom designed A and P1 primers (A primer: 5`-CCATCTCATCCCTGCGTGT*C-3`, P1 primer: 5`-CCACTACGCCTCCGCTTT*C-3`, Eurofins Genomics) and 24 μL adapter-ligated library for a total volume of 60 μL. The libraries were amplified for 8-15 cycles depending on input and DNA source (fragmented DNA was amplified for an additional 2 cycles). Following amplification a post-amplification 1×SPRI cleanup was performed and the DNA was eluted from the beads in 25 µL ddH_2_O. A detailed description of all steps included in this procedure are described in the Kapa HyperPrep Kit protocol (Roche). After the ligation, the samples were analysed on a 2100 Bioanalyzer instrument (Agilent Genomics) using the DNA 1000 kit for assessment of quality and concentration before target capture. An input of 750 ng is required for optimal capture.

### Design of hybridization probes for target enrichment

The eight genes most frequently mutated in pancreatic cancer were selected for the target gene panel based on previous publications and ICGC data (Supplemental [Table 1](#2jxsxqh))^4,, 5,6^. For oncogenes, only hotspot regions were included in the target, whereas for tumor suppressor genes, all coding exons or the most frequently mutated ones were covered. Only exons occurring in the most abundant transcript isoforms were included. The overall size of the panel was 10 495 bases.

Hybridization probes (RNA) for the SureSelect Target Enrichment System (Agilent Technologies) were designed with the SureDesign web service (Design ID 3042181). Probes overlapping with our target panel were extracted from Agilent’s SureSelect Human All Exon V6 exome capture probe set. In this respect, the default boosting and probe density settings were used. In addition, four probes targeting *CDK2A* exon 2 and 3 were added to enhance capture in this genomic region (Supplemental [Table](#3j2qqm3) 6). In total, the panel contained 487 probes over 15.4 kbp.

*Hybridization blocking*

To avoid nonspecific hybridization of probes to the duplex part of the adapters, we designed additional blocking oligos complementary to this region. The sequences were 5’-GCTCTTCCGATCT-3’ (A-stemblocker) and 5’-GATCGGAAGAGC-3’ (P1-stemblocker). The two 3’end nucleotides were linked by a C3 spacer to avoid elongation.

###

### SureSelect Target Enrichment

Target enrichment was performed using the SureSelect Target Enrichment System for Sequencing on Ion Proton (Agilent Technologies). In brief, 750 ng of each library was concentrated in a vacuum centrifuge at 40°C for 20-30 minutes, and resuspended in 3.4 µL ddH_2_O for a final concentration of 221 ng/μL. For each sample, 4.1 µL of each 100 µM stem blocker (P1-Stemblocker: 5`-GATCGGAAGAGC(SpC3)-3` and A-Stemblocker: 5`-GCTCTTCCGATCT(SpC3)-3`) was dried in a vacuum concentrator before being dissolved in 5.6 µL SureSelect Block Mix per sample. The block mix (5.6 µL) was then added to each library (3.4 µL) to make prepped libraries (9 µL) and the libraries were incubated at 95°C for 5 min. Next, the pre-heated hybridization mixture (13 µL) and the prepared libraries (9 µL) were added stepwise to pre-heated capture libraries (7 µL) in a PCR machine pre-heated to 65°C before each sample (containing 27-29 µL, depending on evaporation) was incubated for 24 hours at 65°C for hybridization of the probes to the target regions. For optimal capture of our small library, the capture probe mixture was diluted 1:20 in nuclease-free water before 2 µL was transferred to a PCR tube containing 5 µL of RNAse Block dilution to make the capture library. Next day, library fragments bound to the probes were captured using magnetic beads (Dynabeads MyOne Streptavidin T1, ThermoFisher) washed with prewarmed (70°C) SureSelect Wash 2 buffer 6 times before they were dissolved in 30 μL of nuclease-free water. A more detailed description of all steps included in the target capture procedure is provided in the SureSelect Target Enrichment System of Sequencing on Ion Proton kit protocol (Agilent Technologies). Following the capture, amplification of the target regions was performed by mixing 25 μL 2× KAPA HiFi Hotstart Ready Mix, 2.5 μL 20 µM primer mix (consisting of custom designed A and P1 primers as described in Supplemental Table 5), 14 μL captured library retained on the streptavidin beads plus 8.5 μL ddH_2_O for a total volume of 50 μL. Amplification was performed by initial denaturation at 95°C for 3 minutes followed by 11 cycles of denaturation at 98°C for 20 seconds, annealing at 60°C for 15 seconds and extension at 72°C for 1 minute before final extension at 72°C for 10 minutes. After amplification of the captured libraries, two post-amplification 1×SPRI cleanups were performed before the final libraries were eluted in 25 µL ddH_2_O. All libraries were then analysed on a 2100 Bioanalyzer instrument (Agilent Genomics) using the High Sensitivity DNA kit (Agilent Genomics) for assessment of library quality and concentration.

### Optimization of the procedure

Several steps in this procedure have been optimized. This includes ligation time and temperature (20°C for 15 minutes, 20°C for 4 hours, 4°C overnight), adapter concentrations, library amplification, stem blocker concentrations (0.5×, 1× and 2× of each stemblocker), capture probe concentrations (1:5, 1:10, 1:15, 1:20, 1:25 and 1:30 dilutions), hybridization temperature (65°C and 67°C) and time (16 and 24 hours) as well as the post-capture washing temperature (65°C and 70°C). All implemented modifications of the protocols are described in detail above.

## Next-generation sequencing

Before sequencing, all libraries were diluted in ddH_2_O to 100 pM. Libraries were then pooled (5-16), and used as a template in the emulsion PCR reaction on the Ion OneTouch™ 2 Instrument (ThermoFisher Scientific) using the Ion PI™ Hi-Q ™ OT2 200 kit (ThermoFisher Scientific). After washing the template-positive ISPs, their quality was assessed on a Qubit™ 2.0 Fluorometer using the Ion Sphere™ Quality Control Kit. Enrichment of the templated-Ion sphere particles (ISPs) was performed if the percentage of templated-ISPs was within the recommended range of 10-25%. Enrichment was performed using the Ion OneTouch™ ES instrument following washing of the enriched ISPs before target sequencing on an Ion Proton instrument using the Ion PI™ Hi-Q ™ Sequencing 200 chemistry (ThermoFisher Scientific). All steps are thoroughly described by the manufacturer in the Ion Torrent User manuals (ThermoFisher Scientific).

## Bioinformatic analysis of next-generation sequencing data

### Pre-processing and tag extraction

An overview of the bioinformatic data processing pipeline is presented in supplemental figure 7. Signal processing, base calling, quality control, adapter trimming, sample barcode de-multiplexing and alignment were performed using the Ion Torrent Server software (version 5.12.), with mainly default settings. Base calling calibration was done using the “Blind calibration” setting and custom adapter sequences were uploaded to the server. All adapter sequences except the molecular tag and the duplex part were removed by the Ion Torrent Server software.

On-target reads were extracted from aligned BAM files, forward and reverse reads separated and converted to FASTQ format with samtools (versions 1.8 and 1.10)^7^. Remaining adapter sequences (duplex part) were removed and the molecular tag sequence extracted with a locally developed Python script called TagXtractor. The script was developed from a previously published script^1^ and is available for download from GitHub (https://github.com/oddmundn/TagXtractor). It was called with the following options: --taglen 6 (tag length was 6 bases), --spacerlen 14 (spacer length was 14 bases), --filtspacer AGCTCTTCCGATCT (the sequence of the adapter sequence to be removed, see supplemental figure 1) --endtrimming 5 (removed 5 bases from each end of every read). Five bases were removed from each end of every read to avoid the sequencing errors frequently occurring close to the fragment ends. The script accepts sequencing errors in the spacer sequence (fixed remaining part of the adapter), except for the three last bases, to ensure that the correct molecular tag is extracted. Typically, less than 0,5% of the sequences were discarded due to sequencing errors in the spacer. The molecular tag is being removed from the sequence and added to the read title for later use.

In a typical sequencing file, with 5,511,480 on-target reads, all possible molecular tags were present and the majority of tags was found on 0.01-0.05% of all the reads (Supplemental [Figure](#4i7ojhp) 8a). The most predominant tags were G-rich, probably reflecting some bias in the random nucleotide incorporation during oligonucleotide synthesis. The distribution of tag frequencies was close to a lognormal distribution with median value (¼)^6^ =0.024 % (actual median value was 0.020 %) (Supplemental [Figure](#4i7ojhp) 8B).

### Single strand consensus read generation

Subsequently, the reads were aligned to the reference genome again by BWA-MEM (version 0.7.17^8^), producing aligned and sorted BAM files. Forward reads were aligned to a forward version of the reference genome and reverse reads to a reverse version of the genome. Then, the two (forward and reverse) BAM files were processed separately by a Python script called SSCScreator (<https://github.com/oddmundn/SSCScreator>, version 1.3), which utilized both molecular tag and genome alignment position to collapse reads to single strand consensus sequences. The script was developed from a previous published script named ConsensusMaker^1^ and is available for download at GitHub (URL).

SSCScreator was run with the following options: “--minmem 3 --cutoff 0.7 --filt sn --remove_false --Ncutoff 0.3 --rep_filt 5”. In detail, SSCScreator groups all reads with the same starting position in the genome and the same molecular tag in single strand consensus sequence (SSCS) families. If *minmem* (set to 3) sequences or more share the same starting position and molecular tag, they are subjected to construction of a SSCS. Every position in the SSCS is set to the majority base in that position for the SSCS family, as long as it is present in more than *cutoff* (=0.7) fraction of the reads. If no base fulfills this criterium, an N is inserted in the SSCS. If an SSCS is given more than *Ncutoff* (=0.3) fraction Ns among all positions, the SSCS is discarded. SSCS with tags having homopolymer stretches of 5 or 6 (*--rep_filt*=5) identical bases were discarded. In addition, a base phred quality score is determined for every position of the SSCS, computing the mean base phred quality of the bases used to set the consensus base.

To avoid overestimation of molecular recovery, we also incorporated a functionality in SSCScreator that scans for false SSCS families caused by sequencing errors in the molecular tag (*--remove-false* option). This function compares the molecular tags for a genome position and the neighbouring positions. The occurrence of very similar tags (tag neighbours) are compared to the expected number of such molecular tag neighbours by chance, given the total number of SSCS starting in these positions. If a set of tag neighbours is highly unlikely, the smaller SSCS families are rejected as a false SSCS family and removed. The rationale and extent of such false SSCS families are described in section “Validation of HYTEC-seq bioinformatic pipeline” below.

Finally, SSCScreator outputs all true SSCSs to a FASTQ file, giving every SSCS a name consisting of chromosome position, molecular tag, and the number of SSCS family members. Subsequently, the forward and reverse SSCS files are merged and aligned to the reference genome again with BWA-MEM. The whole process from raw BAM file to SSCS BAM file has been combined in a bash shell script pipeline called *HYTEC-pipeline.sh*, which is available for download at GitHub (https://github.com/oddmundn/HYTEC_pipeline).

Realigned SSCS bam files were subjected to variant calling using the PlasmaMutationDetector2 R package version 1.1.10, which we derived from the PlasmaMutationDetector package^9^.

### Background error profile construction using control plasma samples

At each position not known as SNP using the ExAC release 1.0 database, the coverage was considered together with five background errors: three for each single nucleotide variant (SNV), plus two for potential misalignments, which could be interpreted as coming from a deletion or an insertion (INDEL). Using a set of 60 plasma control samples from healthy individuals, we pooled the observed coverage data and background errors to get position by position the total number of reads and the number of reads associated with each of the five potential errors in a file that we call later on total background errors.

In detail, a target definition file (position_ranges.rda) tagged in known SNP positions was produced from the target BED file (corresponding to supplemental table 1) using the function PrepareLibrary and release 1 of the Exome Aggregation Consortium (ExAC) SNP database. Error profiles for various analyses were produced by the BuildCtrlErrorRate function called on directories of BAM files from normal control samples (normal plasma, normal leukocytes etc.), using the following options: *BuildCtrlErrorRate(ctrl.dir = <dirname>, bai.ext = ".bai",pos_ranges.file = “position_ranges.rda", hotspot.file = <hotspotfilename>, force = TRUE,n.trim=0)*. The hotspot file was constructed using data from the COSMIC database version 82, extracting only variants reported in more than one sample. Variants were filtered against relevant error profiles (patient plasma versus normal plasma etc.) and called using the function DetectPlasmaMutation with the following options: DetectPlasmaMutation(patient.dir = <dirname>, pos_ranges.file = "position_ranges.rda", ber.ctrl.file = “background_error_rate.txt", bai.ext = ".bai", n.trim = 0, force = FALSE, cutoff.sb.hotspot=4, cutoff.sb.ref=0.9, qcutoff.snv=1, qcutoff.indel=1). Only positions with SSCS coverage above 100 and indels longer than 2 bases were considered for variant calling.

### Variant calling: multiplicity control

The reduced error rates caused by using SSCSs led to a simplification of the statistical treatment for variant calling: the filtering only requesting a proper control of the multiplicity. To this end, we process in the following way using Bonferroni correction. Given a (global) false positive error rate 𝛼, which controls the probability to call by mistake a mutation in a healthy patient, we control with the same probability 𝛼/2 the false positives coming from hotspots and from non-hotspots. Given a plasma sample of a patient, we call H the number of hotspots and R of non-hotspots available for this sample in the panel positions. For each of the three single nucleotides, mutations occurring from SNV are tested at level 𝛼/6H at hotspot positions and 𝛼/6R otherwise. Similarly, for insertion and deletion, mutations occurring from INDEL are tested at level 𝛼/4H and 𝛼/4R.

### Variant calling: testing procedure

 Given a patient plasma sample, a given position and a potential mutation, we have now at hand at the considered position: the total coverage (C), the associated background error (B), the *ad-hoc* corrected test level 𝛼*, the patient coverage (**n**) and the patient number of reads associated with the mutation (**m**). Using C and B, we computed an upper bound *p** for the background error rate with confidence level 1-𝛼* using Massart’s inequality for Binomial distributions^10^. Next, we computed the p-value of the test “**m** follows a Binomial distribution of parameters **n** and *q* with q not smaller than *p**”.

The position was declared holding a SNV, if the minimum of the three p-values associated with the three non-reference nucleotype was smaller than the SNV corrected level that is 𝛼/6H or 𝛼/6R, depending on the hotspot status. Similarly, the position was declared holding an INDEL, if the minimum of the two p-values associated with the INDEL was smaller than the INDEL corrected level (𝛼/4H or 𝛼/4R).

It is noticeable that with our approach several mutations could be detected at a single position. Then one has to decide whether returning the most probable, considered as those with smallest corrected p-value, or all variants which have been detected.

**Upper bound for the rate *p* of a Binomial distribution when *B* successes are observed out of *C* trials^10^**

Let us consider *q=B/C* then *p*=q+𝛆*/3 where

*𝛆* = [L(1 - 2*q*) + sqrt(L^2+18C(1-*q*)*q*)] / (2L+9C)

with L = log (1/**𝛂**) is upper bound for *p* with level 1-𝛂. When *B=0*, we assume that *q=*1/*C* log(*C*) or, in other words, that B is smaller than 1 and of order 1/log(*C*) .

### Variant annotation

Variant annotation was supported by the ANNOVAR software version 2018-04-16 with the following options: *table_annovar.pl <query-file> <database-folder> -buildver hg19 –thread 4 -out <outfile> -remove -protocol refGene,cytoBand,exac03,avsnp147,dbnsfp30a,clinvar_20160302 -operation g,r,f,f,f,f -nastring . -polish.*

## Validation of the HYTEC-seq methodological approach

### Importance of removing SSCSs caused by sequencing errors in the molecular tag

Sequencing errors in unique molecular tags (UMIs) are known to produce additional SSCS, differing from the original tag by just a few bases^11^. We investigated whether this was also the case for HYTEC-seq by comparing the mean edit distance (Levenshtein distance according to the editdistance python package) of a typical sample with low cfDNA input (0.63 ng) and one with high DNA input (50 ng) to the mean edit distance for simulated (same number of barcodes in each position as for the real samples) random 6-base barcode duplets (4.15). Only positions where two or more forward SSCSs start were included in these computations. As can be seen in Supplemental Figure 9, the mean edit distance for the low input DNA sample, but not the high-input sample, was significantly lower than expected by random barcode sampling (P<0.001), indicating that sequencing errors in the molecular tags actually generated artifactual SSCS. The problem was more serious for low input samples because of the high sequencing coverage compared to the number of cfDNA input molecules, increasing the likelihood of repeating the same sequencing error (as at least 3 reads were required). When we applied our false SSCS filter as described above, we increased the mean edit distance to almost the same as obtained with randomly selected tags, demonstrating that our filter worked well (Supplemental Figure 9).

### SSCS family size analysis

By SSCS family size we mean the number of raw sequencing reads being collapsed into the same SSCS based on identical orientation, starting position and molecular tag. We randomly selected 5 patient samples with varying amounts of cfDNA input and analyzed their numbers of on-target reads and SSCS family sizes (Supplemental Figure 10).

The number of on-target reads was clearly higher for the high input cfDNA samples (Supplementary Figure 7a). This was done with purpose, as we sequenced fewer samples per chip when having higher cfDNA input amount. The SSCS family sizes also seemed to be larger when increasing sequencing depth (Supplementary Figure 10b). The distribution of SSCS family sizes was generally quite wide and skewed towards small size. Median SSCS family sizes ranged from 9 to 19 in this sample selection, which suggested that our raw sequencing depth was adequate and well adapted to the amount of cfDNA input.

### Molecular recovery analysis

Molecular recovery is the fraction of input DNA fragments that are recovered in the finished library; a measure of the cfDNA capture effectiveness. In the HYTEC-seq method, the molecular recovery is primarily affected by the ligation of adaptors and the target capture. However, the measured molecular recovery is also affected by the number of molecules sequenced, as samples with too low sequencing depth might result in discarded SSCS due to the requirement of ≥3 family members to form a SSCS. We sequenced samples to a target depth of 250,000 on target reads per ng input DNA which we considered sufficient. The results from the sequencing demonstrated a median number of on-target reads per ng input of 262,967 and a median molecular recovery of 22.1% (Mean unique SSCSs/Input DNA fragments) (Supplemental Figure 5). Increasing the sequencing depth also increases the molecular recovery, but the effect is smaller with increasingly higher sequencing depths (note the logarithmic x scale). The level of input DNA had little effect on the molecular recovery (results not shown).

## Determination of analytical sensitivity using commercial and custom-made control samples

Twenty ng of the 100% Multiplex I Wild Type cfDNA Reference Standard (Horizon Discovery) was used for library construction and sequencing using the HYTEC-seq protocol in eight replicates. The resulting SSCS BAM files were used for construction of a background_error_rate.txt file for error correction in the determination of analytical sensitivity, using the PlasmaMutationDetector2 R package. 20 ng of the 5%, 1%, 0.5%, and 0.1% Multiplex I cfDNA Reference Standard (Horizon Discovery) were analysed similarly, in triplicates, and the BAM files were submitted to variant calling using the PlasmaMutationDetector2 package. The 100% Multiplex I Wild Type cfDNA Reference Standards were analysed in parallel as a negative control. The Multiplex I cfDNA Reference standard samples harbored a known p.G12D mutation in the *KRAS* gene at relative variant allele frequencies 5%, 1%, 0.5% and 0.1%, respectively (ref.

product sheet). When using the PlasmaMutationDetector2 *de novo* variant caller, we detected the *KRAS* mutation in all three replicates for the 5% and 1% sample, and in 1/3 replicates for the 0.5% sample (Supplemental Figure 4a). No false positive variants were called. However, SSCS reads containing the *KRAS* mutations were observed in all control samples except the 100% wild-type control. Analysis of 50 ng DNA of the same Horizon control series by the Oncomine Pan-Cancer Assay is further described in the main manuscript (Supplemental Figure 4B).

## Iterated variant calling on normal plasma samples

The 60 normal plasma samples were divided in 6 groups of 10 samples by random sampling. For each sampling of 10, the remaining 50 normal plasma samples were used to generate a background error model for variant calling in the sample. This process was repeated for all 6 groups and all the variants called were collected in a Supplemental Table 2.

**References**

1. Kennedy SR*, et al.* Detecting ultralow-frequency mutations by Duplex Sequencing. *Nat Protoc* **9**, 2586-2606 (2014).

2. Stein C*, et al.* *Discrete Mathematics for Computer Scientists,*. Addison-Wesley Publishing Company (2010).

3. Newman AM*, et al.* Integrated digital error suppression for improved detection of circulating tumor DNA. *Nature biotechnology*, (2016).

4. Witkiewicz AK*, et al.* Whole-exome sequencing of pancreatic cancer defines genetic diversity and therapeutic targets. *Nature communications* **6**, 6744 (2015).

5. Waddell N*, et al.* Whole genomes redefine the mutational landscape of pancreatic cancer. *Nature* **518**, 495-501 (2015).

6. Hudson TJ*, et al.* International network of cancer genome projects. *Nature* **464**, 993-998 (2010).

7. Li H*, et al.* The Sequence Alignment/Map format and SAMtools. *Bioinformatics (Oxford, England)* **25**, 2078-2079 (2009).

8. Li H, Durbin R. Fast and accurate long-read alignment with Burrows-Wheeler transform. *Bioinformatics (Oxford, England)* **26**, 589-595 (2010).

9. Pecuchet N*, et al.* Analysis of Base-Position Error Rate of Next-Generation Sequencing to Detect Tumor Mutations in Circulating DNA. *Clinical chemistry* **62**, 1492-1503 (2016).

10. Massart P. The Tight Constant in the Dvoretzky-Kiefer-Wolfowitz Inequality. *The Annals of Probability* **18**, 1269-1283 (1990).

11. Smith T*, et al.* UMI-tools: modeling sequencing errors in Unique Molecular Identifiers to improve quantification accuracy. *Genome research* **27**, 491-499 (2017).
